# From biophysics to evolutionary genetics: statistical aspects of gene regulation

**DOI:** 10.1186/1471-2105-8-S6-S7

**Published:** 2007-09-27

**Authors:** Michael Lässig

**Affiliations:** 1 Institut für Theoretische Physik, Universität zu Köln, Zülpicher Str., 77, 50937 Köln, Germany

## Abstract

This is an introductory review on how genes interact to produce biological functions. Transcriptional interactions involve the binding of proteins to regulatory DNA. Specific binding sites can be identified by genomic analysis, and these undergo a stochastic evolution process governed by selection, mutations, and genetic drift. We focus on the links between the biophysical function and the evolution of regulatory elements. In particular, we infer fitness landscapes of binding sites from genomic data, leading to a quantitative evolutionary picture of regulation.

## Introduction

Genomic functions often cannot be understood at the level of single genes but require the study of gene networks. This systems biology credo is nearly commonplace by now. Evidence comes from the comparative analysis of entire genomes: Current estimates put, for example, the number of human genes at around 22000, hardly more than the 14000 of the fruit fly, and not even an order of magnitude higher than the 6000 of baker's yeast. The complexity and diversity of higher animals therefore cannot be explained in terms of their gene numbers. If, however, a biological function requires the concerted action of several genes, and conversely, a gene takes part in several functional contexts, an organism may be defined less by its individual genes but by their interactions. The emerging picture of the genome as a strongly interacting system with many degrees of freedom brings new challenges for experiment and theory, many of which are of a statistical nature. And indeed, this picture continues to make the subject attractive to a growing number of statistical physicists.

Genes encode proteins, and proteins perform functions in the cell. Hence, a gene takes part in a biological function only if it is *expressed*, i.e., if the protein produced from it is present in the cell. Genes interact by *regulation*: the protein of one gene can influence the production of protein from another gene. Gene regulation can take place during *transcription*, the process by which the cell reads the information contained in a gene and copies it to messenger RNA (which is subsequently used to make a functional protein). This is the most fundamental level of interactions between genes: the transcription of one gene may be enhanced or reduced by the expression of other genes. Transcriptional regulation is thus a good starting point for theory. We should keep in mind, however, that it is not the only mode of gene interactions. Especially in eukaryotes, additional regulation mechanims involving histones, chromatin, micro-RNAs etc. become relevant, which are just entering the stage of model building. An excellent introduction to the biology of regulation can be found in [1].

This article is a primer on theoretical aspects of gene interactions, and we limit ourselves to transcriptional regulation. Clearly, the subject has rather diverse aspects:

(1) Transcription is a *biophysical *process, which involves the interaction of DNA and proteins. Its regulation takes place through the binding of proteins to DNA at specific loci in the vicinity of the gene to be regulated. Already at this level, this process is rather complex and not yet fully understood. What enables the protein to find one or a few specific functional sites in a genome of up to billions of base pairs, bind there with sufficient strength to influence transcription, and leave again once its task is performed?

(2) Given that the protein can find its functional sites, can we as well? If that is possible, we can predict the specific gene interactions building regulatory networks from sequence data. The analysis of regulatory DNA is a major topic of research in *bioinformatics*, with the aim of identifying statistical characteristics of functional loci and of building search algorithms.

(3) Regulation is also becoming an important part of *evolutionary biology *[2,3]. If regulatory networks are to explain the differentiation of higher animals, there must be efficient modes of evolution for the interactions between genes. At the level of regulary DNA, these modes remain largely to be explored. It is clear, however, that the underlying evolutionary dynamics is the basis of a quantitative understanding of regulatory networks.

All three aspects of regulation contribute to a unified theoretical picture. Key concepts such as the biophysical binding energy, the bioinformatic scoring function, and the evolutionary fitness turn out to be rather deeply related. We will focus on these crosslinks between different fields, which are likely to become important for future research. A challenge for an introductory presentation is the diversity of relevant background material, only a rather ecclectic account of which can be presented here. Yet, I hope it transpires even from this short introduction that present quantitative genomics is an area of science shaped by a remarkable confluence of ideas from different disciplines.

## Biophysics of transcriptional regulation

**Figure 1:**
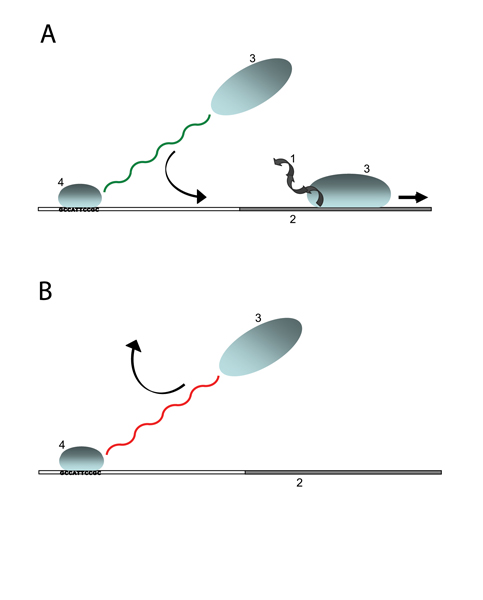
**Transcriptional regulation**. Transcription is the synthesis of messenger RNA (1) whose genetic code is a copy of the coding DNA (2) of a gene, by means of RNA polymerase (3). A transcription factor (4) bound to a DNA target site interacts with RNA polymerase molecules, (a) enhancing or (b) reducing the transcription rate of a nearby gene.

The fundamental step in the regulatory interaction between two genes is a binding process: the protein produced by the first gene acts as a *transcription factor *for the second gene, i.e., it binds to a functional site on the DNA close to the second gene and thereby enhances or suppresses its transcription. Binding sites are short, typically segments of 10 to 15 base pairs in prokaryotes and even shorter segments in eukaryotes. They are primarily located in the *cis-regulatory region *of a gene, which lies just upstream of its protein-coding sequence and extends over hundreds of base pairs in prokaryotes and over thousands of base pairs in eukaryotes. The scenario of transcriptional regulation is sketched in Fig. 1. A transcription factor bound to a functional binding site regulates the downstream gene by recruiting or repelling RNA polymerase. This protein-protein interaction catalyzes or suppresses the process of transcription of the gene. All these binding processes should not be understood as on or off; they happen with certain probabilities, which are determined by the binding energies and the numbers of the molecules involved.

### Factor-DNA binding energies

**Figure 2:**
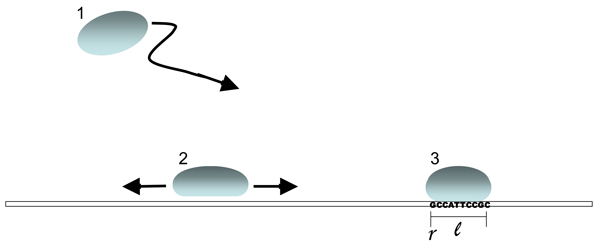
**Thermodynamic states of a transcription factor**. (1) Unbound state, with three-dimensional diffusion. (2) Unspecific bound state, with one-dimensional diffusion along the DNA backbone. (3) Specific bound state. The binding energy depends on the genotype at the binding locus, which has length ℓ and whose position is specified by the coordinate *r*.

The interaction of a transcription factor protein with DNA is two-fold: There is a position-unspecific attraction with energy *E*_*u *_and a specific interaction, whose energy depends on the particular locus where the factor binds. The unspecific part is the electrostatic interaction between the positively charged protein and the negatively charged DNA backbone, while the specific part involves hydrogen bonds between the binding domain of the protein and the nucleotides of the binding locus. A locus is specified by its starting position *r *and its length ℓ (with relevant values ℓ of order 10). The specific binding energy *E*(*r*) depends on ℓ consecutive nucleotides **a **= (*a*_1_,..., *a*_ℓ_) counted downstream from the starting position, the *sequence state *or *genotype *of that locus. Switching between unspecific and specific binding takes place via a conformation change of the factor protein. As a result of these interactions, the factor protein can be in three thermodynamic states as shown in fig. 2: unbound (i.e., freely diffusing), unspecifically bound (i.e., diffusing along the DNA backbone), and specifically bound.

The biophysics of factor-DNA binding has been established in a series of seminal papers [4-7]. More recently, the characteristics of specific binding have been measured for some bacterial transcription factors [8-12]. These can be summarized as follows:

(a) The single nucleotides of a binding locus **a **= (*a*_1_,..., *a*_ℓ_) give approximately independent contributions to the binding energy,

E(a)=∑i=1ℓεi(ai).
 MathType@MTEF@5@5@+=feaafiart1ev1aaatCvAUfKttLearuWrP9MDH5MBPbIqV92AaeXatLxBI9gBamXvP5wqSXMqHnxAJn0BKvguHDwzZbqegyvzYrwyUfgarqqtubsr4rNCHbGeaGqiA8vkIkVAFgIELiFeLkFeLk=iY=Hhbbf9v8qqaqFr0xc9pk0xbba9q8WqFfeaY=biLkVcLq=JHqVepeea0=as0db9vqpepesP0xe9Fve9Fve9GapdbaqaaeGacaGaaiaabeqaamqadiabaaGcbaGaemyrauKaeiikaGccdeGaa8xyaiabcMcaPiabg2da9maaqahabaacciGae4xTdu2aaSbaaSqaaiabdMgaPbqabaGccqGGOaakcqWGHbqydaWgaaWcbaGaemyAaKgabeaakiabcMcaPiabc6caUaWcbaGaemyAaKMaeyypa0JaeGymaedabaGaeS4eHWganiabggHiLdaaaa@5118@

(b) At each position *i*, there is typically one preferred nucleotide ai∗
 MathType@MTEF@5@5@+=feaafiart1ev1aaatCvAUfKttLearuWrP9MDH5MBPbIqV92AaeXatLxBI9gBaebbnrfifHhDYfgasaacH8akY=wiFfYdH8Gipec8Eeeu0xXdbba9frFj0=OqFfea0dXdd9vqai=hGuQ8kuc9pgc9s8qqaq=dirpe0xb9q8qiLsFr0=vr0=vr0dc8meaabaqaciaacaGaaeqabaqabeGadaaakeaacqWGHbqydaqhaaWcbaGaemyAaKgabaGamaiPgEHiQaaaaaa@319A@ with *ε*_*i*_(ai∗
 MathType@MTEF@5@5@+=feaafiart1ev1aaatCvAUfKttLearuWrP9MDH5MBPbIqV92AaeXatLxBI9gBaebbnrfifHhDYfgasaacH8akY=wiFfYdH8Gipec8Eeeu0xXdbba9frFj0=OqFfea0dXdd9vqai=hGuQ8kuc9pgc9s8qqaq=dirpe0xb9q8qiLsFr0=vr0=vr0dc8meaabaqaciaacaGaaeqabaqabeGadaaakeaacqWGHbqydaqhaaWcbaGaemyAaKgabaGamaiPgEHiQaaaaaa@319A@) = min_*a*_*ε*_*i*_(*a*). Hence, there is a unique "ground state" sequence a* = (a1∗
 MathType@MTEF@5@5@+=feaafiart1ev1aaatCvAUfKttLearuWrP9MDH5MBPbIqV92AaeXatLxBI9gBaebbnrfifHhDYfgasaacH8akY=wiFfYdH8Gipec8Eeeu0xXdbba9frFj0=OqFfea0dXdd9vqai=hGuQ8kuc9pgc9s8qqaq=dirpe0xb9q8qiLsFr0=vr0=vr0dc8meaabaqaciaacaGaaeqabaqabeGadaaakeaacqWGHbqydaqhaaWcbaGaeGymaedabaGamaiPgEHiQaaaaaa@312F@,...aℓ∗
 MathType@MTEF@5@5@+=feaafiart1ev1aaatCvAUfKttLearuWrP9MDH5MBPbIqV92AaeXatLxBI9gBaebbnrfifHhDYfgasaacH8akY=wiFfYdH8Gipec8Eeeu0xXdbba9frFj0=OqFfea0dXdd9vqai=hGuQ8kuc9pgc9s8qqaq=dirpe0xb9q8qiLsFr0=vr0=vr0dc8meaabaqaciaacaGaaeqabaqabeGadaaakeaacqWGHbqydaqhaaWcbaGaeS4eHWgabaGamaiPgEHiQaaaaaa@3170@) with minimal binding energy E* = *E*(a*), i.e., with strongest binding.

(c) Mismatches with respect to the minimum-energy sequence involve energy costs *ε*_*i*_(*a*) - *ε*_*i*_(ai∗
 MathType@MTEF@5@5@+=feaafiart1ev1aaatCvAUfKttLearuWrP9MDH5MBPbIqV92AaeXatLxBI9gBaebbnrfifHhDYfgasaacH8akY=wiFfYdH8Gipec8Eeeu0xXdbba9frFj0=OqFfea0dXdd9vqai=hGuQ8kuc9pgc9s8qqaq=dirpe0xb9q8qiLsFr0=vr0=vr0dc8meaabaqaciaacaGaaeqabaqabeGadaaakeaacqWGHbqydaqhaaWcbaGaemyAaKgabaGamaiPgEHiQaaaaaa@319A@) ≈ 1 - 3 *k*_*B*_*T *per nucleotide.

(d) There is an energy difference *E*_*u *_- E* ~15 *k*_*B*_*T *between unspecific and strongest specific binding. Experimental data for the binding energies *ε*_*i*_(*a*) are known only for a few transcription factors.

Approximate values for these energies can also be inferred from nucleotide frequencies in functional binding sites [10]. A promising recent approach is to infer binding energies from large-throughput expression data [13]. For order-of-magnitude estimates, one often uses the so-called two-state approximation [7], which is homogeneous in the nucleotide positions and distinguishes only between match and mismatch:

εi(a)−εi(ai∗)={εif ai≠ai∗0if ai=ai∗
 MathType@MTEF@5@5@+=feaafiart1ev1aaatCvAUfKttLearuWrP9MDH5MBPbIqV92AaeXatLxBI9gBaebbnrfifHhDYfgasaacH8akY=wiFfYdH8Gipec8Eeeu0xXdbba9frFj0=OqFfea0dXdd9vqai=hGuQ8kuc9pgc9s8qqaq=dirpe0xb9q8qiLsFr0=vr0=vr0dc8meaabaqaciaacaGaaeqabaqabeGadaaakeaaiiGacqWF1oqzdaWgaaWcbaGaemyAaKgabeaakiabcIcaOiabdggaHjabcMcaPiabgkHiTiab=v7aLnaaBaaaleaacqWGPbqAaeqaaOGaeiikaGIaemyyae2aa0baaSqaaiabdMgaPbqaaiadasQHxiIkaaGccqGGPaqkcqGH9aqpdaGabaqaauaabaqaciaaaeaacqWF1oqzaeaacqqGPbqAcqqGMbGzcqqGGaaicqWGHbqydaWgaaWcbaGaemyAaKgabeaakiabgcMi5kabdggaHnaaDaaaleaacqWGPbqAaeaacWaGKA4fIOcaaaGcbaGaeGimaadabaGaeeyAaKMaeeOzayMaeeiiaaIaemyyae2aaSbaaSqaaiabdMgaPbqabaGccqGH9aqpcqWGHbqydaqhaaWcbaGaemyAaKgabaGamaiPgEHiQaaaaaaakiaawUhaaaaa@5BCC@

with *ε *≈ 2*k*_*B*_*T *. In this approximation, the binding energy of a sequence **a **is simply related to the *Hamming distance d*(**a**, a*), i.e., the number of nucleotide mismatches between **a **and a*,

*E*(**a**) = E* + *ε*·*d*(**a**, a*).

### Energy distribution in the genome

**Figure 3:**
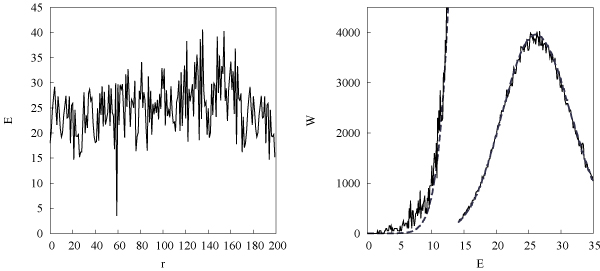
**Transcription factor binding energies of the E. coli genome**. (a) Energy "landscape" *E*(*r*) for specific binding of the CRP factor at 200 consecutive positions *r *in an intergenic region, with a binding site at position 59. (b) Count histogram *W*_dat_(*E*) with energy bins of width 0.1 obtained from all intergenic regions, together with the distribution *W*_0_(*E*) for a random sequence (dashed line, shown with a 30 fold zoom into the region *E *< 14). From [16].

Fig. 3(a) shows the sequence of energy values *E*(*r*) found in a segment of the *E. coli *genome for a specific transcription factor, the cAMP response protein (CRP) This "energy landscape" looks quite random, i.e., consecutive energy values are approximately uncorrelated. The distribution *W*_dat_(*E*) of energies over the entire noncoding part of the *E. coli *genome is shown in fig. 3(b). We can compare this with the distribution *W*_0_(*E*) obtained from a random sequence with the same nucleotide frequencies (i.e., from a scrambled genome). According to eq. (1), the binding energy *E *is then a sum of independent random variables *ε*_*i*_, and its distribution becomes approximately Gaussian by the law of large numbers. Fig. 3(b) shows that the actual distribution *W*_dat_(*E*) is indeed of the same form as *W*_0_(*E*) for most energies. However, a closer look at the low-energy tail of the distribution shows that there are significantly more strong binding sites than expected from a random sequence [14-16]. So at least some of them are there not by chance but for a reason.

### Search kinetics

All three thermodynamic modes of a factor molecule – free diffusion, unspecific binding, and specific binding – are important for the search kinetics towards a functional site [4-6]. The unspecific attraction causes the transcription factor to be bound to DNA with a finite probability, i.e., a given molecule spends about equal amounts of time on and off the DNA backbone. Hence, the search process is a mixture of effectively one-dimensional diffusion along the DNA backbone and three-dimensional diffusion in the surrounding medium. This proves more efficient than purely one- or three-dimensional diffusion. In the 1D mode, the factor diffuses in a flat energy landscape if it is in the conformation of unspecific binding, or in the landscape *E*(*r*) if it is in the conformation of specific binding. In this way, it can sample the low-energy part of the landscape *E*(*r*) while avoiding its barriers. The main obstacles on its way to a functional site are spurious binding sites, which have a low energy *E*(*r*) by chance and act as traps. We lack a completely satisfactory picture of the search kinetics, which is an area of current research [14,17]. However, this process proves to be remarkably fast. Typical search times are less than a minute, i.e., substantially shorter than typical functional intervals in a cell cycle of at least minutes. Therefore, the regulatory effect of a site is related to its probability of binding a factor molecule at equilibrium, which can be evaluated by standard thermodynamics.

### Thermodynamics of factor binding

We start with the idealized but instructive problem of a single factor protein interacting with a genome of length *L *≫ 1, which contains a single functional site, while the rest of the sequence is random. Since the protein is bound to the DNA with a probability of about 1/2, we neglect the unbound state for the subsequent probability estimates and study only the bound protein, which is at equilibrium between specific and unspecific binding. At each position *r*, the likelihood of these two states is given by the Boltzmann factors exp [-*E*(*r*)/*k*_*B*_*T*] and exp [-*E*_*u*_/*k*_*B*_*T*], respectively. Hence, the partition function for a single protein has the form

Z=∑r=1Le−E(r)/kBT+Le−Eu/kBT.
 MathType@MTEF@5@5@+=feaafiart1ev1aaatCvAUfKttLearuWrP9MDH5MBPbIqV92AaeXatLxBI9gBaebbnrfifHhDYfgasaacH8akY=wiFfYdH8Gipec8Eeeu0xXdbba9frFj0=OqFfea0dXdd9vqai=hGuQ8kuc9pgc9s8qqaq=dirpe0xb9q8qiLsFr0=vr0=vr0dc8meaabaqaciaacaGaaeqabaqabeGadaaakeaacqWGAbGwcqGH9aqpdaaeWbqaaiabbwgaLnaaCaaaleqabaGaeyOeI0IaemyrauKaeiikaGIaemOCaiNaeiykaKIaei4la8Iaem4AaS2aaSbaaWqaaiabdkeacbqabaWccqWGubavaaGccqGHRaWkcqWGmbatcqqGLbqzdaahaaWcbeqaaiabgkHiTiabdweafnaaBaaameaacqWG1bqDaeqaaSGaei4la8Iaem4AaS2aaSbaaWqaaiabdkeacbqabaWccqWGubavaaaabaGaemOCaiNaeyypa0JaeGymaedabaGaemitaWeaniabggHiLdGccqGGUaGlaaa@4DE1@

The functional site, which is assumed to be positioned at *r *= *r*_*f*_, must have a low specific binding energy *E *= *E*(*r*_*f*_). We now single out this position and write

Z=e−E/kBT+∑r≠rfe−E(r)/kBT+Le−Eu/kBT≈e−E/kBT+Z0,
 MathType@MTEF@5@5@+=feaafiart1ev1aaatCvAUfKttLearuWrP9MDH5MBPbIqV92AaeXatLxBI9gBaebbnrfifHhDYfgasaacH8akY=wiFfYdH8Gipec8Eeeu0xXdbba9frFj0=OqFfea0dXdd9vqai=hGuQ8kuc9pgc9s8qqaq=dirpe0xb9q8qiLsFr0=vr0=vr0dc8meaabaqaciaacaGaaeqabaqabeGadaaakeaafaqaaeGadaaabaGaemOwaOfabaGaeyypa0dabaGaeeyzau2aaWbaaSqabeaacqGHsislcqWGfbqrcqGGVaWlcqWGRbWAdaWgaaadbaGaemOqaieabeaaliabdsfaubaakiabgUcaRmaaqafabaGaeeyzau2aaWbaaSqabeaacqGHsislcqWGfbqrcqGGOaakcqWGYbGCcqGGPaqkcqGGVaWlcqWGRbWAdaWgaaadbaGaemOqaieabeaaliabdsfaubaakiabgUcaRiabdYeamjabbwgaLnaaCaaaleqabaGaeyOeI0Iaemyrau0aaSbaaWqaaiabdwha1bqabaWccqGGVaWlcqWGRbWAdaWgaaadbaGaemOqaieabeaaliabdsfaubaaaeaacqWGYbGCcqGHGjsUcqWGYbGCdaWgaaadbaGaemOzaygabeaaaSqab0GaeyyeIuoaaOqaaaqaaiabgIKi7cqaaiabbwgaLnaaCaaaleqabaGaeyOeI0IaemyrauKaei4la8Iaem4AaS2aaSbaaWqaaiabdkeacbqabaWccqWGubavaaGccqGHRaWkcqWGAbGwdaWgaaWcbaGaeGimaadabeaakiabcYcaSaaaaaa@65D6@

where *Z*_0 _is the partition function of a completely random sequence. The probability of the factor being bound specifically at the functional site is then

p(E)=e−E/kBTZ=11+e(E−F0)/kBT,
 MathType@MTEF@5@5@+=feaafiart1ev1aaatCvAUfKttLearuWrP9MDH5MBPbIqV92AaeXatLxBI9gBaebbnrfifHhDYfgasaacH8akY=wiFfYdH8Gipec8Eeeu0xXdbba9frFj0=OqFfea0dXdd9vqai=hGuQ8kuc9pgc9s8qqaq=dirpe0xb9q8qiLsFr0=vr0=vr0dc8meaabaqaciaacaGaaeqabaqabeGadaaakeaacqWGWbaCcqGGOaakcqWGfbqrcqGGPaqkcqGH9aqpdaWcaaqaaiabbwgaLnaaCaaaleqabaGaeyOeI0IaemyrauKaei4la8Iaem4AaS2aaSbaaWqaaiabdkeacbqabaWccqWGubavaaaakeaacqWGAbGwaaGaeyypa0ZaaSaaaeaacqaIXaqmaeaacqaIXaqmcqGHRaWkcqqGLbqzdaahaaWcbeqaaiabcIcaOiabdweafjabgkHiTiabdAeagnaaBaaameaacqaIWaamaeqaaSGaeiykaKIaei4la8Iaem4AaS2aaSbaaWqaaiabdkeacbqabaWccqWGubavaaaaaOGaeiilaWcaaa@4C58@

where *F*_0 _= -*k*_*B*_*T *log *Z*_0 _is the free energy for a random genome. Thus, the binding probability depends on the binding energy in a sigmoid way, with a threshold energy *E *= *F*_0 _between strong and weak binding.

This strongly nonlinear dependence is known to physicists as a Fermi function.

It is easy to generalize the thermodynamic formalism to more than one factor molecule. Ignoring the overlap between close sites, each position *r *can be empty or be occupied either by an unspecifically or by a specifically bound factor. Using a chemical potential *σ*, the many-factor partition function can hence be written as

Z(σ)=∏r=1LZ(σ,r),
 MathType@MTEF@5@5@+=feaafiart1ev1aaatCvAUfKttLearuWrP9MDH5MBPbIqV92AaeXatLxBI9gBaebbnrfifHhDYfgasaacH8akY=wiFfYdH8Gipec8Eeeu0xXdbba9frFj0=OqFfea0dXdd9vqai=hGuQ8kuc9pgc9s8qqaq=dirpe0xb9q8qiLsFr0=vr0=vr0dc8meaabaqaciaacaGaaeqabaqabeGadaaakeaacqWGAbGwcqGGOaakiiGacqWFdpWCcqGGPaqkcqGH9aqpdaqeWbqaaiabdQfaAjabcIcaOiab=n8aZjabcYcaSiabdkhaYjabcMcaPiabcYcaSaWcbaGaemOCaiNaeyypa0JaeGymaedabaGaemitaWeaniabg+Givdaaaa@40FA@

where

Z(σ,r)=1+eσ−E(r)/kBT+eσ−Eu/kBT
 MathType@MTEF@5@5@+=feaafiart1ev1aaatCvAUfKttLearuWrP9MDH5MBPbIqV92AaeXatLxBI9gBaebbnrfifHhDYfgasaacH8akY=wiFfYdH8Gipec8Eeeu0xXdbba9frFj0=OqFfea0dXdd9vqai=hGuQ8kuc9pgc9s8qqaq=dirpe0xb9q8qiLsFr0=vr0=vr0dc8meaabaqaciaacaGaaeqabaqabeGadaaakeaacqWGAbGwcqGGOaakiiGacqWFdpWCcqGGSaalcqWGYbGCcqGGPaqkcqGH9aqpcqaIXaqmcqGHRaWkcqqGLbqzdaahaaWcbeqaaiab=n8aZjabgkHiTiabdweafjabcIcaOiabdkhaYjabcMcaPiabc+caViabdUgaRnaaBaaameaacqWGcbGqaeqaaSGaemivaqfaaOGaey4kaSIaeeyzau2aaWbaaSqabeaacqWFdpWCcqGHsislcqWGfbqrdaWgaaadbaGaemyDauhabeaaliabc+caViabdUgaRnaaBaaameaacqWGcbGqaeqaaSGaemivaqfaaaaa@502E@

is a sum over the three thermodynamic states at position *r*: no factor bound, one factor bound specifically or unspecifically. The chemical potential *σ *is determined by the number of factor molecules, *n*, via the relation *n *= (d/d*σ*) log *Z*(*σ*). For actual transcription factor numbers, which are of order 1 - 10^4^, this relation is well approximated by [14]

σ=F0kBT+log⁡n.
 MathType@MTEF@5@5@+=feaafiart1ev1aaatCvAUfKttLearuWrP9MDH5MBPbIqV92AaeXatLxBI9gBaebbnrfifHhDYfgasaacH8akY=wiFfYdH8Gipec8Eeeu0xXdbba9frFj0=OqFfea0dXdd9vqai=hGuQ8kuc9pgc9s8qqaq=dirpe0xb9q8qiLsFr0=vr0=vr0dc8meaabaqaciaacaGaaeqabaqabeGadaaakeaaiiGacqWFdpWCcqGH9aqpdaWcaaqaaiabdAeagnaaBaaaleaacqaIWaamaeqaaaGcbaGaem4AaS2aaSbaaSqaaiabdkeacbqabaGccqWGubavaaGaey4kaSIagiiBaWMaei4Ba8Maei4zaCMaemOBa4MaeiOla4caaa@3CE1@

The functional site is now occupied by a specifically bound factor with probability

p(E)=eσ−E/kBTZ(σ,rf)=11+e(E−F0)/kBT−log⁡n.
 MathType@MTEF@5@5@+=feaafiart1ev1aaatCvAUfKttLearuWrP9MDH5MBPbIqV92AaeXatLxBI9gBaebbnrfifHhDYfgasaacH8akY=wiFfYdH8Gipec8Eeeu0xXdbba9frFj0=OqFfea0dXdd9vqai=hGuQ8kuc9pgc9s8qqaq=dirpe0xb9q8qiLsFr0=vr0=vr0dc8meaabaqaciaacaGaaeqabaqabeGadaaakeaacqWGWbaCcqGGOaakcqWGfbqrcqGGPaqkcqGH9aqpdaWcaaqaaiabbwgaLnaaCaaaleqabaacciGae83WdmNaeyOeI0IaemyrauKaei4la8Iaem4AaS2aaSbaaWqaaiabdkeacbqabaWccqWGubavaaaakeaacqWGAbGwcqGGOaakcqWFdpWCcqGGSaalcqWGYbGCdaWgaaWcbaGaemOzaygabeaakiabcMcaPaaacqGH9aqpdaWcaaqaaiabigdaXaqaaiabigdaXiabgUcaRiabbwgaLnaaCaaaleqabaGaeiikaGIaemyrauKaeyOeI0IaemOray0aaSbaaWqaaiabicdaWaqabaWccqGGPaqkcqGGVaWlcqWGRbWAdaWgaaadbaGaemOqaieabeaaliabdsfaujabgkHiTiGbcYgaSjabc+gaVjabcEgaNjabd6gaUbaaaaGccqGGUaGlaaa@5BDE@

The binding probability – and hence the effects of the functional site on the regulated gene – are thus determined by the binding energy, the number of factor molecules, and on the genomic background (via the free energy *F*_0_). The dependence *p*(*E*) is a Fermi function with threshold energy *E *= *F*_0 _+ *k*_*B*_*T *log *n*, which is shifted with respect to the single-molecule case. Clearly, *p *is also a Fermi function of log *n *at fixed binding energy, with a threshold at log *n *= (*E *- *F*_0_)/*k*_*B*_*T *. If there is more than one functional site in the genome, the calculation remains unaffected as long as their number is much smaller than *n*.

### Sensitivity and genomic design of regulation

The regulatory machinery can be very efficient: in bacteria, it has been shown that single factor molecules can have regulatory effects. We can use eq. (6) to enquire how the cell can reach this high level of sensitivity, following mostly ref. [14]. We assume a minimal genome which has a single functional site of maximum binding strength E* and is otherwise random. If a single factor molecule is to affect regulation, its binding to the functional site must not be overwhelmed by the remainder of the genome. This leads to a criterion on the signal-to-noise ratio of regulatory interactions,

*F*_0 _≳ E*,

which in turn imposes a number of constraints on the design of regulatory DNA:

(a) In a random genome, there must be at most a number of order one minimum-energy binding sites. Estimating the probability to find such a site at a given position as (1/4)^ℓ^, we obtain the condition

*L*(1/4)^ℓ ^≲ 1.

This gives a lower bound on the site length, ℓ ≳ log *L*/log 4. For a bacterial genome (*L *~10^6^), we obtain ℓ ≳ 10, which gives the right length of functional binding sites. However, this bound is not fulfilled in eukaryotes. Indeed, eukaryotic genomes use a different design with groups of adjacent binding sites.

(b) For each minimum-energy site, there are ℓ suboptimal sites of Hamming distance 1 from the minimum-energy sequence. These must not suppress the binding to the minimum-energy site, i.e.,

exp(-E*/*k*_*B*_*T*) ≳ ℓ exp [-(E* + *ε*)/*k*_*B*_*T*]

in the two-state approximation. This gives a lower bound on the binding energy per nucleotide, *ε*/*k*_*B*_*T *≳ log ℓ ≈ 2 - 3.

(c) Finally, the unspecific binding in the entire genome must not suppress the specific binding to a minimum-energy site, i.e.,

exp(-E*/*k*_*B*_*T*) ≳ *L *exp(-*E*_*u*_/*k*_*B*_*T*).

This produces a lower bound on the energy gap between unspecific and optimal specific binding, (*E*_*u *_- E*)/*k*_*B*_*T *≳ log *L *≈ 15.

Quite remarkably, these bounds are fulfilled as approximate equalities in bacteria. Hence, the machinery of transcriptional regulation operates just at the treshold of single-molecule sensitivity, i.e, *F*_0 _≈ E*.

### Programmability and evolvability of regulatory networks

Of course, not every regulatory interaction is equally sensitive. To switch genes on or off, the cell uses the dependencies of the binding probability both on factor numbers and on binding energies. During the cell cycle, the level of *n *can vary over several orders of magnitude, say, between a few and tens of thousands of molecules. At a given value of *n*, the effects on the regulated genes differ since their functional sites have different values of *E*. The binding energies can change on evolutionary time scales by mutations of the site sequence, which leads to regulatory differences between individuals and, ultimately, between species. Both parameters are thus necessary to encode pathways in regulatory networks. This is most flexible if minimum-energy sites are indeed sensitive to a single factor molecule as discussed above. Differential *programmability *as a network design principle [14] thus favors complicated molecular structures with longer binding sites and larger binding energies. However, this competes with the *evolvability *of the system by a stochastic evolution process [18]. We have seen that the single-molecule sensitivity is just marginally reached in bacteria. This indicates that the actual machinery may result from a compromise between programmability and evolvability: binding sites are just complicated enough to work. It also indicates that genomic structures can only be understood from their evolution. This aspect will be developed further below, after we have introduced sequence analysis aspects in the next section.

## Bioinformatics of regulatory DNA

Predicting regulatory interactions between genes is clearly a key problem in bioinformatics, which is as important as the analysis of individual genes and proteins. It is not surprising that this problem is very difficult since, as we have discussed in the previous section, targeting regulatory input in a large genome is a tremendous signal-to-noise problem even for the cell itself. Its solution via the analysis of regulatory DNA requires finding statistical criteria to distinguish between functional binding sites and background sequence. A general introduction to the relevant sequence statistics can be found in ref. [19].

### Markov model for background sequence

We begin by specifying a stochastic model for the nonfunctional segments of intergenic DNA. These are assumed to be Markov sequences with uniform single-nucleotide frequencies *p*_0_(*a*) (*a *= *A*, *C*, *G*, *T*). Hence, the probability of finding a given sequence has the factorized form

P0(a1,...,ak)=∏i=1kp0(ai).
 MathType@MTEF@5@5@+=feaafiart1ev1aaatCvAUfKttLearuWrP9MDH5MBPbIqV92AaeXatLxBI9gBaebbnrfifHhDYfgasaacH8akY=wiFfYdH8Gipec8Eeeu0xXdbba9frFj0=OqFfea0dXdd9vqai=hGuQ8kuc9pgc9s8qqaq=dirpe0xb9q8qiLsFr0=vr0=vr0dc8meaabaqaciaacaGaaeqabaqabeGadaaakeaacqWGqbaudaWgaaWcbaGaeGimaadabeaakiabcIcaOiabdggaHnaaBaaaleaacqaIXaqmaeqaaOGaeiilaWIaeiOla4IaeiOla4IaeiOla4IaeiilaWIaemyyae2aaSbaaSqaaiabdUgaRbqabaGccqGGPaqkcqGH9aqpdaqeWbqaaiabdchaWnaaBaaaleaacqaIWaamaeqaaOGaeiikaGIaemyyae2aaSbaaSqaaiabdMgaPbqabaGccqGGPaqkcqGGUaGlaSqaaiabdMgaPjabg2da9iabigdaXaqaaiabdUgaRbqdcqGHpis1aaaa@4A4E@

This assumption should not be taken too literally. The term "nonfunctional" refers to binding of a particular transcription factor. Intergenic DNA contains plenty of non-random elements with other functions (e.g., binding sites for other factors) or without known function (such as repeat elements). The salient point is, however, that most of intergenic DNA is well approximated by a Markov sequence with respect to binding of a given transcription factor. To make this more precise, we project the distribution *P*_0_(**a**) for segments of length ℓ onto the binding energy *E *as independent variable. Denoting the projected distribution for simplicity with the same letter *P*_0_, we have

P0(E)≡∑aP0(a)δ(E−E(a)).
 MathType@MTEF@5@5@+=feaafiart1ev1aaatCvAUfKttLearuWrP9MDH5MBPbIqV92AaeXatLxBI9gBaebbnrfifHhDYfgasaacH8akY=wiFfYdH8Gipec8Eeeu0xXdbba9frFj0=OqFfea0dXdd9vqai=hGuQ8kuc9pgc9s8qqaq=dirpe0xb9q8qiLsFr0=vr0=vr0dc8meaabaqaciaacaGaaeqabaqabeGadaaakeaacqWGqbaudaWgaaWcbaGaeGimaadabeaakiabcIcaOiabdweafjabcMcaPiabggMi6oaaqafabaGaemiuaa1aaSbaaSqaaiabicdaWaqabaGccqGGOaakieqacqWFHbqycqGGPaqkiiGacqGF0oazcqGGOaakcqWGfbqrcqGHsislcqWGfbqrcqGGOaakcqWFHbqycqGGPaqkcqGGPaqkcqGGUaGlaSqaaiab=fgaHbqab0GaeyyeIuoaaaa@468E@

This distribution is close to the actual genomic distribution *W*_dat_(*E*) for most values of *E*, as we have seen in fig. 3. It is possible to improve the background model by introducing small frequency couplings between neigboring letters [15,16].

### Probabilistic model for functional sites

The sequences **a **= (*a*_1_,..., *a*_ℓ_) at functional sites of a given transcription factor are assumed to be drawn from a different distribution *Q*(**a**). We write this distribution in the form

*Q*(**a**) = *P*_0_(**a**) exp [*S*(**a**)].

The quantity *S*(**a**), which is called the *relative log likelihood score *of the distributions *P*_0 _and *Q*, will turn out to have an important evolutionary meaning as well.

The single-nucleotide distribution *q*_*i*_(*a*) at a given position *i *within functional loci is obtained by summing the full distribution *Q *over all other positions

qi(a)=∑a1,...,ai−1,ai+1,...,aℓQ(a).
 MathType@MTEF@5@5@+=feaafiart1ev1aaatCvAUfKttLearuWrP9MDH5MBPbIqV92AaeXatLxBI9gBaebbnrfifHhDYfgasaacH8akY=wiFfYdH8Gipec8Eeeu0xXdbba9frFj0=OqFfea0dXdd9vqai=hGuQ8kuc9pgc9s8qqaq=dirpe0xb9q8qiLsFr0=vr0=vr0dc8meaabaqaciaacaGaaeqabaqabeGadaaakeaacqWGXbqCdaWgaaWcbaGaemyAaKgabeaakiabcIcaOiabdggaHjabcMcaPiabg2da9maaqafabaGaemyuaeLaeiikaGccbeGae8xyaeMaeiykaKcaleaacqWGHbqydaWgaaadbaGaeGymaedabeaaliabcYcaSiabc6caUiabc6caUiabc6caUiabcYcaSiabdggaHnaaBaaameaacqWGPbqAcqGHsislcqaIXaqmaeqaaSGaeiilaWIaemyyae2aaSbaaWqaaiabdMgaPjabgUcaRiabigdaXaqabaWccqGGSaalcqGGUaGlcqGGUaGlcqGGUaGlcqGGSaalcqWGHbqydaWgaaadbaGaeS4eHWgabeaaaSqab0GaeyyeIuoakiabc6caUaaa@5334@

The set of these marginal distributions, *q*_*i*_(*a*) (*i *= 1,..., ℓ; *a *= *A*, *C*, *G*, *T*) is called the *position weight matrix *for binding sites of a given factor [20]. If the score function is additive in the nucleotide positions, S(a)=∑i=1ℓsi(ai)
 MathType@MTEF@5@5@+=feaafiart1ev1aaatCvAUfKttLearuWrP9MDH5MBPbIqV92AaeXatLxBI9gBaebbnrfifHhDYfgasaacH8akY=wiFfYdH8Gipec8Eeeu0xXdbba9frFj0=OqFfea0dXdd9vqai=hGuQ8kuc9pgc9s8qqaq=dirpe0xb9q8qiLsFr0=vr0=vr0dc8meaabaqaciaacaGaaeqabaqabeGadaaakeaacqWGtbWucqGGOaakieqacqWFHbqycqGGPaqkcqGH9aqpdaaeWaqaaiabdohaZnaaBaaaleaacqWGPbqAaeqaaOGaeiikaGIaemyyae2aaSbaaSqaaiabdMgaPbqabaGccqGGPaqkaSqaaiabdMgaPjabg2da9iabigdaXaqaaiabloriSbqdcqGHris5aaaa@3FF6@, the *Q *distribution has a factorized form, Q(a)=∏i=1ℓqi(ai)
MathType@MTEF@5@5@+=feaafiart1ev1aaatCvAUfKttLearuWrP9MDH5MBPbIqV92AaeXatLxBI9gBaebbnrfifHhDYfgasaacH8akY=wiFfYdH8Gipec8Eeeu0xXdbba9frFj0=OqFfea0dXdd9vqai=hGuQ8kuc9pgc9s8qqaq=dirpe0xb9q8qiLsFr0=vr0=vr0dc8meaabaqaciaacaGaaeqabaqabeGadaaakeaacqWGrbqucqGGOaakieqacqWFHbqycqGGPaqkcqGH9aqpdaqeWaqaaiabdghaXnaaBaaaleaacqWGPbqAaeqaaOGaeiikaGIaemyyae2aaSbaaSqaaiabdMgaPbqabaGccqGGPaqkaSqaaiabdMgaPjabg2da9iabigdaXaqaaiabloriSbqdcqGHpis1aaaa@3FDD@ with

*q*_*i*_(*a*) = *p*_0_(*a*) exp [*s*_*i*_(*a*)].

This additivity assumption is made in most of the existing literature since the position weight matrix (18) can be inferred from a sample of known functional site sequences, which in turn determines directly the single nucleotide scores (19). This scoring is the basis for a number of site prediction methods in single species and by cross-species analysis; see, e.g., refs. [20-24].

Here we treat functional sites as coherent statistical units and do not make the assumption of additivity of the score function [16]. As will be discussed in the next section, functionality imposes correlations between the nucleotide frequencies within a functional site, preventing factorization of the *Q *distribution. Of course, it is not possible to reconstruct the full distribution *Q*(**a**), which lives on a 4^ℓ^-dimensional sequence space, from a limited sample of experimentally known functional sites. However, we can again project this distribution onto the binding energy as independent variable, *Q*(*E*) = *∑*_**a **_*Q*(**a**)*δ*(*E *- *E*(**a**)). Since all regulatory effects of a functional site depend on its sequence *a *only via the binding energy, we can also write the score as a function of the energy, *S*(**a**) = *S*(*E*(**a**)) (this will become obvious in the next section). Hence, the relationship (17) has the same form for the projected distributions,

*Q*(*E*) = *P*_0_(*E*) exp [*S*(*E*)].

### Bayesian model for genomic loci

Assuming that functional loci are distributed randomly with a small probability *λ*, we now combine the models for background sequence and for functional sites into a model for the full distribution of sequences **a **in intergenic DNA,

*W*(**a**) = (1 - *λ*) *P*_0_(**a**) + *λ Q*(**a**).

(At the moment, we are ignoring the possible overlap between functional sites). In the language of statistics, this is a probabilistic model with *hidden variables*. The output of this model consists of pairs (*m*, **a**): First, the model variable *m *is randomly drawn, labelling a locus as nonfunctional (*m *= 0) with probability 1 - *λ *or as functional (*m *= 1) with probability *λ*. Then the sequence is drawn from the corresponding distribution *P*_0_(**a**) or *Q*(**a**). However, only the sequence counts **a **are available data. The "hidden" variable *m *can be inferred from the data in a probabilistic way using Bayes' formula, which expresses the joint probability distribution of data and model in terms of its conditional and its marginal distributions

prob(**a**, *m*) = prob(**a**|*m*) prob(*m*) = prob(*m*|**a**) prob(**a**)

with prob(**a**) = ∑_*m*_prob(**a**|*m*)prob(*m*). We can solve for the conditional probability of the model for given data **a**,

prob(m|a)=prob(a|m)prob(m)∑mprob(a|m)prob(m).
 MathType@MTEF@5@5@+=feaafiart1ev1aaatCvAUfKttLearuWrP9MDH5MBPbIqV92AaeXatLxBI9gBaebbnrfifHhDYfgasaacH8akY=wiFfYdH8Gipec8Eeeu0xXdbba9frFj0=OqFfea0dXdd9vqai=hGuQ8kuc9pgc9s8qqaq=dirpe0xb9q8qiLsFr0=vr0=vr0dc8meaabaqaciaacaGaaeqabaqabeGadaaakeaacqqGWbaCcqqGYbGCcqqGVbWBcqqGIbGycqGGOaakcqWGTbqBcqGG8baFieqacqWFHbqycqGGPaqkcqGH9aqpdaWcaaqaaiabbchaWjabbkhaYjabb+gaVjabbkgaIjabcIcaOiab=fgaHjabcYha8jabd2gaTjabcMcaPiabbchaWjabbkhaYjabb+gaVjabbkgaIjabcIcaOiabd2gaTjabcMcaPaqaamaaqababaGaeeiCaaNaeeOCaiNaee4Ba8MaeeOyaiMaeiikaGIae8xyaeMaeiiFaWNaemyBa0MaeiykaKIaeeiCaaNaeeOCaiNaee4Ba8MaeeOyaiMaeiikaGIaemyBa0MaeiykaKcaleaacqWGTbqBaeqaniabggHiLdaaaOGaeiOla4caaa@6548@

For the probability of functionality, *ρ_f_*(**a**) = prob(*m *= 1|**a**), this formula reads

ρf(a)=λQ(a)W(a)=11+exp⁡[−S(a)+log⁡1−λλ].
 MathType@MTEF@5@5@+=feaafiart1ev1aaatCvAUfKttLearuWrP9MDH5MBPbIqV92AaeXatLxBI9gBaebbnrfifHhDYfgasaacH8akY=wiFfYdH8Gipec8Eeeu0xXdbba9frFj0=OqFfea0dXdd9vqai=hGuQ8kuc9pgc9s8qqaq=dirpe0xb9q8qiLsFr0=vr0=vr0dc8meaabaqaciaacaGaaeqabaqabeGadaaakeaaiiGacqWFbpGCdaWgaaWcbaGaemOzaygabeaakiabcIcaOGqabiab+fgaHjabcMcaPiabg2da9maalaaabaGae83UdWMaemyuaeLaeiikaGIae4xyaeMaeiykaKcabaGaem4vaCLaeiikaGIae4xyaeMaeiykaKcaaiabg2da9maalaaabaGaeGymaedabaGaeGymaeJaey4kaSIagiyzauMaeiiEaGNaeiiCaa3aamWaaeaacqGHsislcqWGtbWucqGGOaakcqGFHbqycqGGPaqkcqGHRaWkcyGGSbaBcqGGVbWBcqGGNbWzdaWcaaqaaiabigdaXiabgkHiTiab=T7aSbqaaiab=T7aSbaaaiaawUfacaGLDbaaaaGaeiOla4caaa@5858@

The dependence on *S *has again the form of a Fermi function. Its threshold value *S *= log [(1 - *λ*)/*λ*] separates sequences that are more likely to be functional or more likely to be background.

The full Bayesian model (21) can again be projected onto the energy variable,

*W*(*E*) = (1 - *λ*)*P*_0_(*E*) + *λ Q*(*E*).

**Figure 4:**
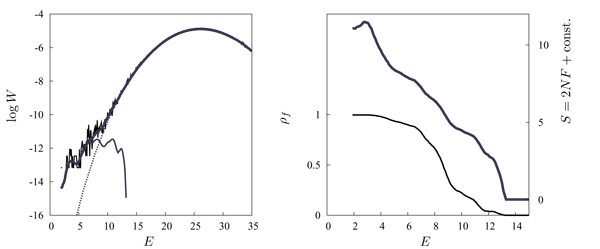
**Bayesian model for regulatory DNA and score function**. (a) Energy count histogram *W*_dat_(*E*) for CRP sites in *E. coli *as in fig. 3 (log scale), model distribution *W*(*E*) (thick line), and its decomposition (25) into background component (1 - *λ*)*P*_0_(*E*) (thin dashed line) and component *λ Q*(*E*) (*E *<*E*_*s *_≈ 13) of functional sites (thin solid line). (b) Log-likelihood score *S*(*E*) = log [*Q*(*E*)/*P*_0_(*E*)] (shifted by a constant, thick line) and probability of functionality *ρ*_*f*_(*E*) (thin line). From [16].

In this form, it can be tested against genomic data [16]. To plot the distributions *P*_0_, *Q*, and *W *as functions of *E*, we use eq. (1) with an energy matrix *ε*_*i*_(*a*) = *ε*_0 _log [*q*_*i*_(*a*)/*p*_0_(*a*)] estimated from the position weight matrix up to an overall constant *ε*_0 _[10]. For our example of the CRP transcription factor, the distribution *Q*(*E*) can be estimated from the about 50 known binding sites in the *E. coli *genome. Using this *Q *distribution and a probability of functionality *λ *≈ 6 × 10^-4^, the full distribution *W*(*E*) produces an excellent fit of the count histogram *W*_dat_(*E*) over the entire range of energies; see fig. 4(a). The log likelihood score function *S*(*E*) = log [*Q*(*E*)/*P*_0_(*E*)] is shown in fig. 4(b), shifted such that the curve has its zero at a point *E*_*s *_≈ 13 beyond which binding becomes negligible.

The resulting probability of functionality *ρf*(*E*) as given by eq. (24) is also shown in fig. 4(b). This indicates the dilemma for the prediction of individual binding sites based on sequence data from a single species. Many functional sites have energies in the "twilight" region between the ensembles *λQ *and (1 - *λ*)*P*_0_, where *ρf *takes values around 1/2. Hence, depending on the energy cutoff chosen, any prediction is torn between many false negatives or many false positives.

### Dynamic programming and sequence analysis

**Figure 5:**
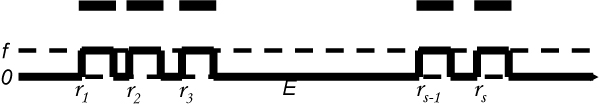
**Analysis of regulatory sequences**. A configuration of *s *nonoverlapping binding sites is given by the sequence of left initial positions **r**_*f *_= (*r*_1_,..., *r*_*s*_) (with *r*_*ν*+1 _- *r*_*ν *_≥ ℓ for *ν *= 1, 2,..., *s *- 1). It can be associated with a path *m*(*r*) which takes the values *m *= 1 at the nucleotide positions of binding sites and *m *= 0 elsewhere. Dynamic programming algorithms based on a Bayesian model (27) of genomic sequences assign to each site configuration a probability of occurence *ρ *(**r**|*a*_1_,..., *a*_*L*_) for given sequence data *a*_1_,..., *a*_*L*_; see eq. (29).

It is straightforward to generalize the Bayesian approach to longer segments of intergenic DNA, which are covered by an unknown number *s *of non-overlapping functional sites as shown in fig. 5[22]. The hidden variables are now the sequence of left initial positions **r**_*f *_≡ (*r*_1_,..., *r*_*s*_) of the functional sites (with the no-overlap constraint *r*_*ν*+1 _≥ *r*_*ν *_+ ℓ for *ν *= 1,..., *s *- 1). The full sequence distribution in a segment of length *L *has the form

WL(a1,...,aL)=Z−1∑rfλ˜WL(a1,...,aL|rf),
 MathType@MTEF@5@5@+=feaafiart1ev1aaatCvAUfKttLearuWrP9MDH5MBPbIqV92AaeXatLxBI9gBaebbnrfifHhDYfgasaacH8akY=wiFfYdH8Gipec8Eeeu0xXdbba9frFj0=OqFfea0dXdd9vqai=hGuQ8kuc9pgc9s8qqaq=dirpe0xb9q8qiLsFr0=vr0=vr0dc8meaabaqaciaacaGaaeqabaqabeGadaaakeaacqWGxbWvdaWgaaWcbaGaemitaWeabeaakiabcIcaOiabdggaHnaaBaaaleaacqaIXaqmaeqaaOGaeiilaWIaeiOla4IaeiOla4IaeiOla4IaeiilaWIaemyyae2aaSbaaSqaaiabdYeambqabaGccqGGPaqkcqGH9aqpcqWGAbGwdaahaaWcbeqaaiabgkHiTiabigdaXaaakmaaqafabaacciGaf83UdWMbaGaacqWGxbWvdaWgaaWcbaGaemitaWeabeaaaeaaieqacqGFYbGCdaWgaaadbaGaemOzaygabeaaaSqab0GaeyyeIuoakiabcIcaOiabdggaHnaaBaaaleaacqaIXaqmaeqaaOGaeiilaWIaeiOla4IaeiOla4IaeiOla4IaeiilaWIaemyyae2aaSbaaSqaaiabdYeambqabaGccqGG8baFcqGFYbGCdaWgaaWcbaGaemOzaygabeaakiabcMcaPiabcYcaSaaa@58B5@

where *Z *is a normalization factor, λ˜
 MathType@MTEF@5@5@+=feaafiart1ev1aaatCvAUfKttLearuWrP9MDH5MBPbIqV92AaeXatLxBI9gBaebbnrfifHhDYfgasaacH8akY=wiFfYdH8Gipec8Eeeu0xXdbba9frFj0=OqFfea0dXdd9vqai=hGuQ8kuc9pgc9s8qqaq=dirpe0xb9q8qiLsFr0=vr0=vr0dc8meaabaqaciaacaGaaeqabaqabeGadaaakeaaiiGacuWF7oaBgaacaaaa@2E76@ = *λ *+ *O*(*λ*^2^) is a weight factor for each functional locus (the negligible correction terms originate from the no-overlap constraint), and *W*_*L*_(*a*_1_,..., *a*_*L*_|**r**_*f*_) is the sequence distribution for given positions of functional loci,

WL(a1,...,aL|rf)=     p0(a1)...p0(ar1−1)∏ν=1sQ(arν,...,arν+ℓ−1)p0(arν+ℓ)...p0(arν+1−1)=     p0(a1)...p0(aL)exp⁡[∑ν=1sS(arν,...,arν+ℓ−1)]
 MathType@MTEF@5@5@+=feaafiart1ev1aaatCvAUfKttLearuWrP9MDH5MBPbIqV92AaeXatLxBI9gBaebbnrfifHhDYfgasaacH8akY=wiFfYdH8Gipec8Eeeu0xXdbba9frFj0=OqFfea0dXdd9vqai=hGuQ8kuc9pgc9s8qqaq=dirpe0xb9q8qiLsFr0=vr0=vr0dc8meaabaqaciaacaGaaeqabaqabeGadaaakqGabeGaaWaaaqxabaGaem4vaC1aaSbaaSqaaiabdYeambqabaGccqGGOaakcqWGHbqydaWgaaWcbaGaeGymaedabeaakiabcYcaSiabc6caUiabc6caUiabc6caUiabcYcaSiabdggaHnaaBaaaleaacqWGmbataeqaaOGaeiiFaWhcbeGae8NCai3aaSbaaSqaaiabdAgaMbqabaGccqGGPaqkcqGH9aqpaeaacaWLjaGaaCzcaiabdchaWnaaBaaaleaacqaIWaamaeqaaOGaeiikaGIaemyyae2aaSbaaSqaaiabigdaXaqabaGccqGGPaqkcqGGUaGlcqGGUaGlcqGGUaGlcqWGWbaCdaWgaaWcbaGaeGimaadabeaakiabcIcaOiabdggaHnaaBaaaleaacqWGYbGCdaWgaaadbaGaeGymaedabeaaliabgkHiTiabigdaXaqabaGccqGGPaqkdaqeWbqaaiabdgfarjabcIcaOiabdggaHnaaBaaaleaacqWGYbGCdaWgaaadbaacciGae4xVd4gabeaaaSqabaGccqGGSaalcqGGUaGlcqGGUaGlcqGGUaGlcqGGSaalcqWGHbqydaWgaaWcbaGaemOCai3aaSbaaWqaaiab+17aUbqabaWccqGHRaWkcqWItecBcqGHsislcqaIXaqmaeqaaOGaeiykaKIaemiCaa3aaSbaaSqaaiabicdaWaqabaGccqGGOaakcqWGHbqydaWgaaWcbaGaemOCai3aaSbaaWqaaiab+17aUbqabaWccqGHRaWkcqWItecBaeqaaOGaeiykaKIaeiOla4IaeiOla4IaeiOla4IaemiCaa3aaSbaaSqaaiabicdaWaqabaGccqGGOaakcqWGHbqydaWgaaWcbaGaemOCai3aaSbaaWqaaiab+17aUjabgUcaRiabigdaXaqabaWccqGHsislcqaIXaqmaeqaaOGaeiykaKIaeyypa0daleaacqGF9oGBcqGH9aqpcqaIXaqmaeaacqWGZbWCa0Gaey4dIunaaOqaaiaaxMaacaWLjaGaaCzcaiabdchaWnaaBaaaleaacqaIWaamaeqaaOGaeiikaGIaemyyae2aaSbaaSqaaiabigdaXaqabaGccqGGPaqkcqGGUaGlcqGGUaGlcqGGUaGlcqWGWbaCdaWgaaWcbaGaeGimaadabeaakiabcIcaOiabdggaHnaaBaaaleaacqWGmbataeqaaOGaeiykaKIagiyzauMaeiiEaGNaeiiCaa3aamWaaeaadaaeWbqaaiabdofatjabcIcaOiabdggaHnaaBaaaleaacqWGYbGCdaWgaaadbaGae4xVd4gabeaaaSqabaGccqGGSaalcqGGUaGlcqGGUaGlcqGGUaGlcqGGSaalcqWGHbqydaWgaaWcbaGaemOCai3aaSbaaWqaaiab+17aUbqabaWccqGHRaWkcqWItecBcqGHsislcqaIXaqmaeqaaOGaeiykaKcaleaacqGF9oGBcqGH9aqpcqaIXaqmaeaacqWGZbWCa0GaeyyeIuoaaOGaay5waiaaw2faaaaaaa@C1D0@

with *r*_*n*+1 _= *L *+ 1. The sum over sequences **r**_*f *_of arbitrary length *s *seems formidable at first, but *W*_*L *_is easy to compute from the recursion

*W*_*r*_(*a*_1_,..., *a*_*r*_) = (1 - λ^
 MathType@MTEF@5@5@+=feaafiart1ev1aaatCvAUfKttLearuWrP9MDH5MBPbIqV92AaeXatLxBI9gBaebbnrfifHhDYfgasaacH8akY=wiFfYdH8Gipec8Eeeu0xXdbba9frFj0=OqFfea0dXdd9vqai=hGuQ8kuc9pgc9s8qqaq=dirpe0xb9q8qiLsFr0=vr0=vr0dc8meaabaqaciaacaGaaeqabaqabeGadaaakeaaiiGacuWF7oaBgaqcaaaa@2E77@)*p*_0_(*a*_*r*_)*W*_*r*-1_(*a*_1_,..., *a*_*r*-1_) + λ˜
 MathType@MTEF@5@5@+=feaafiart1ev1aaatCvAUfKttLearuWrP9MDH5MBPbIqV92AaeXatLxBI9gBaebbnrfifHhDYfgasaacH8akY=wiFfYdH8Gipec8Eeeu0xXdbba9frFj0=OqFfea0dXdd9vqai=hGuQ8kuc9pgc9s8qqaq=dirpe0xb9q8qiLsFr0=vr0=vr0dc8meaabaqaciaacaGaaeqabaqabeGadaaakeaaiiGacuWF7oaBgaacaaaa@2E76@*Q*(*a*_*r*-ℓ+1_,..., *a*_*r*_) *W*_*r*-ℓ_(*a*_1_,..., *a*_*r*-ℓ_)

with the initial condition *W*_0 _= 1 and λ^
 MathType@MTEF@5@5@+=feaafiart1ev1aaatCvAUfKttLearuWrP9MDH5MBPbIqV92AaeXatLxBI9gBaebbnrfifHhDYfgasaacH8akY=wiFfYdH8Gipec8Eeeu0xXdbba9frFj0=OqFfea0dXdd9vqai=hGuQ8kuc9pgc9s8qqaq=dirpe0xb9q8qiLsFr0=vr0=vr0dc8meaabaqaciaacaGaaeqabaqabeGadaaakeaaiiGacuWF7oaBgaqcaaaa@2E77@ = λ˜
 MathType@MTEF@5@5@+=feaafiart1ev1aaatCvAUfKttLearuWrP9MDH5MBPbIqV92AaeXatLxBI9gBaebbnrfifHhDYfgasaacH8akY=wiFfYdH8Gipec8Eeeu0xXdbba9frFj0=OqFfea0dXdd9vqai=hGuQ8kuc9pgc9s8qqaq=dirpe0xb9q8qiLsFr0=vr0=vr0dc8meaabaqaciaacaGaaeqabaqabeGadaaakeaaiiGacuWF7oaBgaacaaaa@2E76@ + *O*(λ˜
 MathType@MTEF@5@5@+=feaafiart1ev1aaatCvAUfKttLearuWrP9MDH5MBPbIqV92AaeXatLxBI9gBaebbnrfifHhDYfgasaacH8akY=wiFfYdH8Gipec8Eeeu0xXdbba9frFj0=OqFfea0dXdd9vqai=hGuQ8kuc9pgc9s8qqaq=dirpe0xb9q8qiLsFr0=vr0=vr0dc8meaabaqaciaacaGaaeqabaqabeGadaaakeaaiiGacuWF7oaBgaacaaaa@2E76@^2^). This type of recursion relation is usually called a *dynamic programming algorithm *in computer science. In physics, it is known as a *transfer matrix*, and the sum (27) is recognized as the corresponding discrete path integral in imaginary time *r*, if we interpret **r**_*f *_as encoding a path *m*(*r*) that takes the value *m *= 1 at the nucleotide positions *r*_*ν*_,..., *r*_*ν *_+ ℓ - 1 (*ν *= 1,..., *s*) within functional loci and *m *= 0 otherwise (see fig. 5). Both concepts prove very useful also in more general problems of sequence alignment.

In analogy to (24), the probability of a set **r**_*f *_of functional loci for given sequence data is

ρ(rf|a1,...,aL)=WL(a1,...,aL|rf)WL(a1,...,aL).
 MathType@MTEF@5@5@+=feaafiart1ev1aaatCvAUfKttLearuWrP9MDH5MBPbIqV92AaeXatLxBI9gBaebbnrfifHhDYfgasaacH8akY=wiFfYdH8Gipec8Eeeu0xXdbba9frFj0=OqFfea0dXdd9vqai=hGuQ8kuc9pgc9s8qqaq=dirpe0xb9q8qiLsFr0=vr0=vr0dc8meaabaqaciaacaGaaeqabaqabeGadaaakeaaiiGacqWFbpGCcqGGOaakieqacqGFYbGCdaWgaaWcbaGaemOzaygabeaakiabcYha8jabdggaHnaaBaaaleaacqaIXaqmaeqaaOGaeiilaWIaeiOla4IaeiOla4IaeiOla4IaeiilaWIaemyyae2aaSbaaSqaaiabdYeambqabaGccqGGPaqkcqGH9aqpdaWcaaqaaiabdEfaxnaaBaaaleaacqWGmbataeqaaOGaeiikaGIaemyyae2aaSbaaSqaaiabigdaXaqabaGccqGGSaalcqGGUaGlcqGGUaGlcqGGUaGlcqGGSaalcqWGHbqydaWgaaWcbaGaemitaWeabeaakiabcYha8jab+jhaYnaaBaaaleaacqWGMbGzaeqaaOGaeiykaKcabaGaem4vaC1aaSbaaSqaaiabdYeambqabaGccqGGOaakcqWGHbqydaWgaaWcbaGaeGymaedabeaakiabcYcaSiabc6caUiabc6caUiabc6caUiabcYcaSiabdggaHnaaBaaaleaacqWGmbataeqaaOGaeiykaKcaaiabc6caUaaa@600C@

The most likely set rf∗
 MathType@MTEF@5@5@+=feaafiart1ev1aaatCvAUfKttLearuWrP9MDH5MBPbIqV92AaeXatLxBI9gBaebbnrfifHhDYfgasaacH8akY=wiFfYdH8Gipec8Eeeu0xXdbba9frFj0=OqFfea0dXdd9vqai=hGuQ8kuc9pgc9s8qqaq=dirpe0xb9q8qiLsFr0=vr0=vr0dc8meaabaqaciaacaGaaeqabaqabeGadaaakeaaieqacqWFYbGCdaqhaaWcbaGaemOzaygabaGamaiMgEHiQaaaaaa@31B0@ can be obtained by the following "backward" algorithm: Given the sequence (*W*_1_,..., *W*_*L*_) obtained from the "forward" recursion (28), we can decide for every point *r *whether it is more likely to be a background position or the endpoint of a functional locus, ignoring all sequence information from positions > *r*. This depends on whether the leading contribution to *W*_*r *_comes from the first or second term on the r.h.s. of (28) and defines the local optimum model m*(*r*). The global optimum set of functional loci respecting the no-overlap constraint is then rf∗
 MathType@MTEF@5@5@+=feaafiart1ev1aaatCvAUfKttLearuWrP9MDH5MBPbIqV92AaeXatLxBI9gBaebbnrfifHhDYfgasaacH8akY=wiFfYdH8Gipec8Eeeu0xXdbba9frFj0=OqFfea0dXdd9vqai=hGuQ8kuc9pgc9s8qqaq=dirpe0xb9q8qiLsFr0=vr0=vr0dc8meaabaqaciaacaGaaeqabaqabeGadaaakeaaieqacqWFYbGCdaqhaaWcbaGaemOzaygabaGamaiMgEHiQaaaaaa@31B0@ = {*r|b*(*r*) = 1}, where *b*(*r*) is given by the recursion *b*(*r*) = ℓ if *b*(*r *+ 1) ≤ 1 &m*(*r*) = 1 and *b*(*r*) = max(*b*(*r *+ 1) - 1, 0) otherwise, with the initial condition *b*(*L *+ 1) = 0.

The Bayesian model can easily be extended to sequences containing several types of binding sites, which bind different transcription factors and are distinguished by their *Q *distributions. Dynamic programming algorithms can thus predict the likely coverage of a sequence with binding sites of known type [22]. This is the first step in extending the statistical analysis from single binding sites to entire regions of regulatory DNA. Indeed, models of this kind have been applied successfully to predict regulatory elements in eukaryotes, which typically consist of functional groups of adjacent binding sites. In the algorithms currently used, however, the scoring in (27) is strictly additive for groups of non-overlapping binding sites: it does not take into account dependencies between the sites within one functional group or overlapping sites within one sequence.

## Evolution of regulatory DNA

In the statistical picture developed so far, background sequences and functional sites are reduced to ensembles *P*_0 _and *Q*. This picture is incomplete in two ways. On one hand, it is quite disconnected from the biophysical aspects discussed before: the specific function of binding sites hardly enters the standard formalism of position weight matrices. On the other hand, there is not yet any notion of time and dynamics. Sequences change by various mutation processes, and the observed sequence ensembles derive from this evolutionary dynamics. The evolution of functional loci is fundamentally different from that of background sequence: it is subject to *natural selection*, that is, the fitness of an organism depends on its genotype **a **at a functional locus via the effects on the regulated gene. At this point, the biophysics of binding enters the evolution of functional sequences [25-27]. Moreover, it becomes clear that the statistical framework has to be extended from individual sequences to distributions of genotypes in a population. In this section, we develop an evolutionary picture of regulatory DNA, from which we obtain expressions for the sequence ensembles *P*_0_, *Q*, and the score function *S*. The next four paragraphs are a self-contained introduction to the underlying concepts of population genetics.

### Deterministic population dynamics and fitness

We start by describing the evolution of a large population, which contains individuals of different genotypes **a**. Each genotype is assumed to produce a specific *phenotype*, which may influence the reproductive success of the individuals carrying it. With respect to factor binding, the phenotype can be associated with the binding energy *E*(**a**), since presumably all organismic effects of a locus depend on its genotype only via the binding energy. However, the discussion in the following paragraphs is more general.

We first assume that the subpopulations of a given genotype reproduce separately, i.e., there neither transitions between genotypes through mutations nor (in a sexually reproducing population) mixing through genomic recombination. Writing the dynamics of the subpopulations in the form of simple growth laws,

ddtNa(t)=Fa(t)Na(t),
 MathType@MTEF@5@5@+=feaafiart1ev1aaatCvAUfKttLearuWrP9MDH5MBPbIqV92AaeXatLxBI9gBaebbnrfifHhDYfgasaacH8akY=wiFfYdH8Gipec8Eeeu0xXdbba9frFj0=OqFfea0dXdd9vqai=hGuQ8kuc9pgc9s8qqaq=dirpe0xb9q8qiLsFr0=vr0=vr0dc8meaabaqaciaacaGaaeqabaqabeGadaaakeaadaWcaaqaaiabbsgaKbqaaiabbsgaKjabdsha0baacqWGobGtdaWgaaWcbaacbeGae8xyaegabeaakiabcIcaOiabdsha0jabcMcaPiabg2da9iabdAeagnaaBaaaleaacqWFHbqyaeqaaOGaeiikaGIaemiDaqNaeiykaKIaemOta40aaSbaaSqaaiab=fgaHbqabaGccqGGOaakcqWG0baDcqGGPaqkcqGGSaalaaa@43FA@

defines the (Malthusian) *fitness F*_**a**_(*t*) of each genotype. For notational simplicity, we now limit ourselves to the case of just two genotypes **a **and **b**, where (30) can be written as growth laws for the total population size *N*(*t*) = *N*_**a**_(*t*) + *N*_**b**_(*t*) and for the population fraction *x*(*t*) = *N*_**b**_(*t*)/*N *(*t*) of genotype **b**,

ddtN(t)=F¯(t)N(t),
 MathType@MTEF@5@5@+=feaafiart1ev1aaatCvAUfKttLearuWrP9MDH5MBPbIqV92AaeXatLxBI9gBaebbnrfifHhDYfgasaacH8akY=wiFfYdH8Gipec8Eeeu0xXdbba9frFj0=OqFfea0dXdd9vqai=hGuQ8kuc9pgc9s8qqaq=dirpe0xb9q8qiLsFr0=vr0=vr0dc8meaabaqaciaacaGaaeqabaqabeGadaaakeaadaWcaaqaaiabbsgaKbqaaiabbsgaKjabdsha0baacqWGobGtcqGGOaakcqWG0baDcqGGPaqkcqGH9aqpcuWGgbGrgaqeaiabcIcaOiabdsha0jabcMcaPiabd6eaojabcIcaOiabdsha0jabcMcaPiabcYcaSaaa@3F91@

ddtx(t)=ΔFab(t)x(t)[1−x(t)],
 MathType@MTEF@5@5@+=feaafiart1ev1aaatCvAUfKttLearuWrP9MDH5MBPbIqV92AaeXatLxBI9gBaebbnrfifHhDYfgasaacH8akY=wiFfYdH8Gipec8Eeeu0xXdbba9frFj0=OqFfea0dXdd9vqai=hGuQ8kuc9pgc9s8qqaq=dirpe0xb9q8qiLsFr0=vr0=vr0dc8meaabaqaciaacaGaaeqabaqabeGadaaakeaadaWcaaqaaiabbsgaKbqaaiabbsgaKjabdsha0baacqWG4baEcqGGOaakcqWG0baDcqGGPaqkcqGH9aqpcqGHuoarcqWGgbGrdaWgaaWcbaacbeGae8xyaeMae8NyaigabeaakiabcIcaOiabdsha0jabcMcaPiabdIha4jabcIcaOiabdsha0jabcMcaPiabcUfaBjabigdaXiabgkHiTiabdIha4jabcIcaOiabdsha0jabcMcaPiabc2faDjabcYcaSaaa@4D51@

with F¯
 MathType@MTEF@5@5@+=feaafiart1ev1aaatCvAUfKttLearuWrP9MDH5MBPbIqV92AaeXatLxBI9gBaebbnrfifHhDYfgasaacH8akY=wiFfYdH8Gipec8Eeeu0xXdbba9frFj0=OqFfea0dXdd9vqai=hGuQ8kuc9pgc9s8qqaq=dirpe0xb9q8qiLsFr0=vr0=vr0dc8meaabaqaciaacaGaaeqabaqabeGadaaakeaacuWGgbGrgaqeaaaa@2DD9@(*t*) ≡ [1 - *x*(*t*)]*F*_**a**_(*t*) + *x*(*t*)*F*_**b**_(*t*) and Δ*F*_**ab**_(*t*) ≡ *F*_**b**_(*t*) - *F*_**a**_(*t*). This decomposition is useful since the overall growth rate F¯
 MathType@MTEF@5@5@+=feaafiart1ev1aaatCvAUfKttLearuWrP9MDH5MBPbIqV92AaeXatLxBI9gBaebbnrfifHhDYfgasaacH8akY=wiFfYdH8Gipec8Eeeu0xXdbba9frFj0=OqFfea0dXdd9vqai=hGuQ8kuc9pgc9s8qqaq=dirpe0xb9q8qiLsFr0=vr0=vr0dc8meaabaqaciaacaGaaeqabaqabeGadaaakeaacuWGgbGrgaqeaaaa@2DD9@(*t*) is often strongly time-dependent due to external conditions (e.g., seasonality), while fitness differences, which reflect intrinsic properties of the phenotypes, are more stable. Different genotypes coexisting in a population frequently produce the same or very similar phenotypes and thus have equal fitness (Δ*F*_**ab **_= 0).

**Figure 6:**
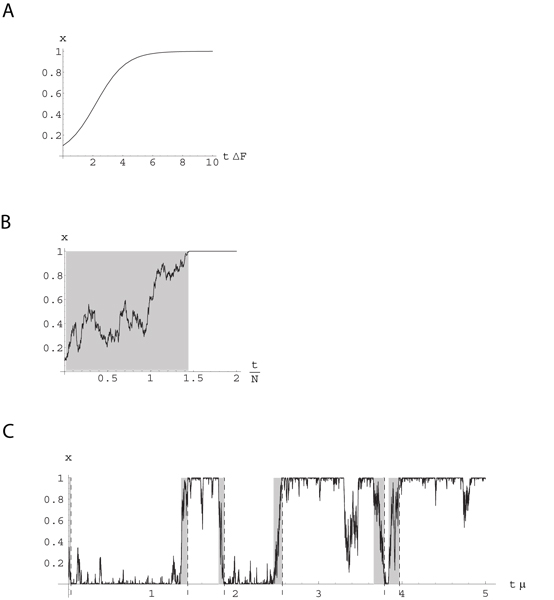
**Evolution of genotype composition *x*(*t*)**. (a) Deterministic evolution with fitness difference Δ*F*_**ab **_> 0, leading to certain fixation of genotype **b **(time is shown in units of *τ*_*d *_= 1/Δ*F*_**ab**_). (b) Stochastic evolution with selection and genetic drift, leading to fixation of one of the genotypes. The time to fixation (grey shading) is of order *τ*_*s *_(*N*Δ*F*_**ab **_= 0.5, time is shown in units of *N*). (c) Stochastic evolution with selection, genetic drift, and mutations in the regime *Nμ *≪ 1, leading to a substitution dynamics with rates *u*_**a**→**b **_and *u*_**b**→**a **_given by (49). Substitution events are marked by dashed lines. The typical time between initial mutation and fixation (grey shading) for a given substitution, *τ*_*s*_, is much shorter than the time between subsequent substitutions, 1/*u*_**a**→**b **_resp. 1/*u*_**b**→**a **_(*N*Δ*F*_**ab **_= 0.5, *Nμ *= 0.05, time is shown in units of 1/*μ*).

Assuming Δ*F*_**ab **_to be constant over the time of observation, the solution of eq. (32) is the evolutionary trajectory

x(t)=x0exp⁡[ΔFab(t−t0)]1−x0(exp⁡[ΔFab(t−t0)]−1)
 MathType@MTEF@5@5@+=feaafiart1ev1aaatCvAUfKttLearuWrP9MDH5MBPbIqV92AaeXatLxBI9gBaebbnrfifHhDYfgasaacH8akY=wiFfYdH8Gipec8Eeeu0xXdbba9frFj0=OqFfea0dXdd9vqai=hGuQ8kuc9pgc9s8qqaq=dirpe0xb9q8qiLsFr0=vr0=vr0dc8meaabaqaciaacaGaaeqabaqabeGadaaakeaacqWG4baEcqGGOaakcqWG0baDcqGGPaqkcqGH9aqpdaWcaaqaaiabdIha4naaBaaaleaacqaIWaamaeqaaOGagiyzauMaeiiEaGNaeiiCaaNaei4waSLaeyiLdqKaemOray0aaSbaaSqaaGqabiab=fgaHjab=jgaIbqabaGccqGGOaakcqWG0baDcqGHsislcqWG0baDdaWgaaWcbaGaeGimaadabeaakiabcMcaPiabc2faDbqaaiabigdaXiabgkHiTiabdIha4naaBaaaleaacqaIWaamaeqaaOGaeiikaGIagiyzauMaeiiEaGNaeiiCaaNaei4waSLaeyiLdqKaemOray0aaSbaaSqaaiab=fgaHjab=jgaIbqabaGccqGGOaakcqWG0baDcqGHsislcqWG0baDdaWgaaWcbaGaeGimaadabeaakiabcMcaPiabc2faDjabgkHiTiabigdaXiabcMcaPaaaaaa@6244@

with the initial condition *x*(*t*_0_) = *x*_0_, shown in fig. 6(a). For Δ*F*_**ab **_≠ 0, the fixed points of this dynamics are the monomorphic population states *x *= 0, and *x *= 1, of which *x *= 1 is stable for Δ*F*_**ab **_> 1 and *x *= 0 for Δ*F*_**ab **_< 1. The approach to the stationary state takes place on a characteristic time scale *τ*_*d *_= 1/Δ*F*_**ab**_.

In the important case of *neutral evolution *(Δ*F*_**ab **_= 0), the evolutionary outcome remains indefinite. These results, which can readily be generalized to more than two phenotypes, are a simple version of Fisher's *fundamental theorem of natural selection*: any population with initially coexisting phenotypes of different fitness will evolve towards a state where only the fittest phenotype is present.

Fisher's theorem seems to prove the popularized Darwinian notion of the "survival of the fittest". However, it rests on very restrictive assumptions that are never fulfilled in a natural population. The deterministic growth law (32) neglects mutations and recombinations, as well as the reproductive fluctuations present in any population due to its finite number of individuals. These other evolutionary forces have to be incorporated in our theoretical picture before we can even define fitness as a measurable quantity and before the theory can address the important case of neutral evolution.

### Stochastic dynamics and genetic drift

Stochastic fluctuations of the reproduction process in a large but finite population have been studied extensively in population genetics, see [28,29]. They are called *genetic drift*, an unfortunate name which may falsely suggest a deterministic effect. To take these fluctuations into account, we replace eq. (30) by a stochastic growth law,

ddtNa(t)=Fa(t)Na(t)+χa(t),
 MathType@MTEF@5@5@+=feaafiart1ev1aaatCvAUfKttLearuWrP9MDH5MBPbIqV92AaeXatLxBI9gBaebbnrfifHhDYfgasaacH8akY=wiFfYdH8Gipec8Eeeu0xXdbba9frFj0=OqFfea0dXdd9vqai=hGuQ8kuc9pgc9s8qqaq=dirpe0xb9q8qiLsFr0=vr0=vr0dc8meaabaqaciaacaGaaeqabaqabeGadaaakeaadaWcaaqaaiabbsgaKbqaaiabbsgaKjabdsha0baacqWGobGtdaWgaaWcbaacbeGae8xyaegabeaakiabcIcaOiabdsha0jabcMcaPiabg2da9iabdAeagnaaBaaaleaacqWFHbqyaeqaaOGaeiikaGIaemiDaqNaeiykaKIaemOta40aaSbaaSqaaiab=fgaHbqabaGccqGGOaakcqWG0baDcqGGPaqkcqGHRaWkiiGacqGFhpWydaWgaaWcbaGae8xyaegabeaakiabcIcaOiabdsha0jabcMcaPiabcYcaSaaa@4B39@

where *χ*_**a**_(*t*) are Gaussian random variables with χa(t)¯=0
 MathType@MTEF@5@5@+=feaafiart1ev1aaatCvAUfKttLearuWrP9MDH5MBPbIqV92AaeXatLxBI9gBaebbnrfifHhDYfgasaacH8akY=wiFfYdH8Gipec8Eeeu0xXdbba9frFj0=OqFfea0dXdd9vqai=hGuQ8kuc9pgc9s8qqaq=dirpe0xb9q8qiLsFr0=vr0=vr0dc8meaabaqaciaacaGaaeqabaqabeGadaaakeaadaqdaaqaaGGaciab=D8aJnaaBaaaleaaieqacqGFHbqyaeqaaOGaeiikaGIaemiDaqNaeiykaKcaaiabg2da9iabicdaWaaa@3518@ and

χa(t)χb(t′)¯=Na(t)δ(t−t′)δa,b.
 MathType@MTEF@5@5@+=feaafiart1ev1aaatCvAUfKttLearuWrP9MDH5MBPbIqV92AaeXatLxBI9gBaebbnrfifHhDYfgasaacH8akY=wiFfYdH8Gipec8Eeeu0xXdbba9frFj0=OqFfea0dXdd9vqai=hGuQ8kuc9pgc9s8qqaq=dirpe0xb9q8qiLsFr0=vr0=vr0dc8meaabaqaciaacaGaaeqabaqabeGadaaakeaadaqdaaqaaGGaciab=D8aJnaaBaaaleaaieqacqGFHbqyaeqaaOGaeiikaGIaemiDaqNaeiykaKIae83Xdm2aaSbaaSqaaiab+jgaIbqabaGccqGGOaakcuWG0baDgaqbaiabcMcaPaaacqGH9aqpcqWGobGtdaWgaaWcbaGae4xyaegabeaakiabcIcaOiabdsha0jabcMcaPiab=r7aKjabcIcaOiabdsha0jabgkHiTiqbdsha0zaafaGaeiykaKIae8hTdq2aaSbaaSqaaiab+fgaHjabcYcaSiab+jgaIbqabaGccqGGUaGlaaa@4DA2@

This form of noise is simply due to the law of large numbers, and the continuum dynamics (34) emerges as an effective large-*N *description for a plethora of discrete evolution models, which are defined at the level of individuals and have finite generation times. In the application to real populations, *N *has to be interpreted as the so-called *effective population size*, which can be inferred from genome data and is in general smaller than the actual population size.

In the case of two genotypes, eq. (34) can again be projected onto the population fraction *x*,

ddtx(t)=ΔFab(t)x(t)[1−x(t)]+χx(t),
 MathType@MTEF@5@5@+=feaafiart1ev1aaatCvAUfKttLearuWrP9MDH5MBPbIqV92AaeXatLxBI9gBaebbnrfifHhDYfgasaacH8akY=wiFfYdH8Gipec8Eeeu0xXdbba9frFj0=OqFfea0dXdd9vqai=hGuQ8kuc9pgc9s8qqaq=dirpe0xb9q8qiLsFr0=vr0=vr0dc8meaabaqaciaacaGaaeqabaqabeGadaaakeaadaWcaaqaaiabbsgaKbqaaiabbsgaKjabdsha0baacqWG4baEcqGGOaakcqWG0baDcqGGPaqkcqGH9aqpcqGHuoarcqWGgbGrdaWgaaWcbaacbeGae8xyaeMae8NyaigabeaakiabcIcaOiabdsha0jabcMcaPiabdIha4jabcIcaOiabdsha0jabcMcaPiabcUfaBjabigdaXiabgkHiTiabdIha4jabcIcaOiabdsha0jabcMcaPiabc2faDjabgUcaRGGaciab+D8aJnaaBaaaleaacqWG4baEaeqaaOGaeiikaGIaemiDaqNaeiykaKIaeiilaWcaaa@54C2@

where *χ*_*x*_(*t*) = (*∂x*/*∂N*_**a**_)*χ*_**a**_(*t*) + (*∂x*/*∂N*_**b**_)*χ*_**b**_(*t*) are Gaussian random variables with zero mean and

χx(t)χx(t′)¯=x(1−x)Nδ(t−t′).
 MathType@MTEF@5@5@+=feaafiart1ev1aaatCvAUfKttLearuWrP9MDH5MBPbIqV92AaeXatLxBI9gBaebbnrfifHhDYfgasaacH8akY=wiFfYdH8Gipec8Eeeu0xXdbba9frFj0=OqFfea0dXdd9vqai=hGuQ8kuc9pgc9s8qqaq=dirpe0xb9q8qiLsFr0=vr0=vr0dc8meaabaqaciaacaGaaeqabaqabeGadaaakeaadaqdaaqaaGGaciab=D8aJnaaBaaaleaacqWG4baEaeqaaOGaeiikaGIaemiDaqNaeiykaKIae83Xdm2aaSbaaSqaaiabdIha4bqabaGccqGGOaakcuWG0baDgaqbaiabcMcaPaaacqGH9aqpdaWcaaqaaiabdIha4jabcIcaOiabigdaXiabgkHiTiabdIha4jabcMcaPaqaaiabd6eaobaacqWF0oazcqGGOaakcqWG0baDcqGHsislcuWG0baDgaqbaiabcMcaPiabc6caUaaa@4AAA@

This dynamics produces stochastic evolutionary trajectories *x*(*t*) as shown in fig. 6(b). To capture their statistics, we convert the Langevin equation (36) into a Fokker-Planck equation for the probability distribution of the genotype composition [28,30],

∂∂tP(x,t)=12N∂2∂x2x(1−x)P(x,t)−ΔFab(t)∂∂xx(1−x)P(x,t).
 MathType@MTEF@5@5@+=feaafiart1ev1aaatCvAUfKttLearuWrP9MDH5MBPbIqV92AaeXatLxBI9gBaebbnrfifHhDYfgasaacH8akY=wiFfYdH8Gipec8Eeeu0xXdbba9frFj0=OqFfea0dXdd9vqai=hGuQ8kuc9pgc9s8qqaq=dirpe0xb9q8qiLsFr0=vr0=vr0dc8meaabaqaciaacaGaaeqabaqabeGadaaakeaadaWcaaqaaiabgkGi2cqaaiabgkGi2kabdsha0baacqqGqbaucqGGOaakcqWG4baEcqGGSaalcqWG0baDcqGGPaqkcqGH9aqpdaWcaaqaaiabigdaXaqaaiabikdaYiabd6eaobaadaWcaaqaaiabgkGi2oaaCaaaleqabaGaeGOmaidaaaGcbaGaeyOaIyRaemiEaG3aaWbaaSqabeaacqaIYaGmaaaaaOGaemiEaGNaeiikaGIaeGymaeJaeyOeI0IaemiEaGNaeiykaKIaeeiuaaLaeiikaGIaemiEaGNaeiilaWIaemiDaqNaeiykaKIaeyOeI0IaeyiLdqKaemOray0aaSbaaSqaaGqabiab=fgaHjab=jgaIbqabaGccqGGOaakcqWG0baDcqGGPaqkdaWcaaqaaiabgkGi2cqaaiabgkGi2kabdIha4baacqWG4baEcqGGOaakcqaIXaqmcqGHsislcqWG4baEcqGGPaqkcqqGqbaucqGGOaakcqWG4baEcqGGSaalcqWG0baDcqGGPaqkcqGGUaGlaaa@6B3D@

The mathematical subtlety of this equation lies in the *x*-dependent diffusion "constant" *x*(1 - *x*)/2*N*, which reflects the multiplicative nature of the reproduction process. As a consequence, the two monomorphic population states *x *= 0 and *x *= 1 are also fixed points also of the stochastic dynamics. Any evolutionary trajectory *x*(*t*) will eventually lead to one of these states with probability 1; this is called the *fixation *of the corresponding genotype in the population. In other words, the Fokker-Planck equation (38) describes diffusion in the interval (0, 1) with *absorbing boundaries*. There is a family of stationary states

P
 MathType@MTEF@5@5@+=feaafiart1ev1aaatCvAUfKttLearuWrP9MDH5MBPbIqV92AaeXatLxBI9gBaebbnrfifHhDYfgasaacH8akY=wiFfYdH8Gipec8Eeeu0xXdbba9frFj0=OqFfea0dXdd9vqai=hGuQ8kuc9pgc9s8qqaq=dirpe0xb9q8qiLsFr0=vr0=vr0dc8meaabaqaciaacaGaaeqabaqabeGadaaakeaacqqGqbauaaa@2DD3@(*x*) = (1 - *φ*)*δ*(*x*) + *φδ *(1 - *x*),

parametrized by the *fixation probability φ *of genotype **b**. The value of *φ *depends on the initial condition *x*_0 _and can be computed by solving the backward diffusion equation

∂∂tP(x,t|x0,t0)=x0(1−x0)(12N∂2∂x02+ΔFab(t)∂∂x0)P(x,t|x0,t0).
 MathType@MTEF@5@5@+=feaafiart1ev1aaatCvAUfKttLearuWrP9MDH5MBPbIqV92AaeXatLxBI9gBaebbnrfifHhDYfgasaacH8akY=wiFfYdH8Gipec8Eeeu0xXdbba9frFj0=OqFfea0dXdd9vqai=hGuQ8kuc9pgc9s8qqaq=dirpe0xb9q8qiLsFr0=vr0=vr0dc8meaabaqaciaacaGaaeqabaqabeGadaaakeaadaWcaaqaaiabgkGi2cqaaiabgkGi2kabdsha0baacqqGqbaucqGGOaakcqWG4baEcqGGSaalcqWG0baDcqGG8baFcqWG4baEdaWgaaWcbaGaeGimaadabeaakiabcYcaSiabdsha0naaBaaaleaacqaIWaamaeqaaOGaeiykaKIaeyypa0JaemiEaG3aaSbaaSqaaiabicdaWaqabaGccqGGOaakcqaIXaqmcqGHsislcqWG4baEdaWgaaWcbaGaeGimaadabeaakiabcMcaPmaabmaabaWaaSaaaeaacqaIXaqmaeaacqaIYaGmcqWGobGtaaWaaSaaaeaacqGHciITdaahaaWcbeqaaiabikdaYaaaaOqaaiabgkGi2kabdIha4naaDaaaleaacqaIWaamaeaacqaIYaGmaaaaaOGaey4kaSIaeyiLdqKaemOray0aaSbaaSqaaGqabiab=fgaHjab=jgaIbqabaGccqGGOaakcqWG0baDcqGGPaqkdaWcaaqaaiabgkGi2cqaaiabgkGi2kabdIha4naaBaaaleaacqaIWaamaeqaaaaaaOGaayjkaiaawMcaaiabbcfaqjabcIcaOiabdIha4jabcYcaSiabdsha0jabcYha8jabdIha4naaBaaaleaacqaIWaamaeqaaOGaeiilaWIaemiDaq3aaSbaaSqaaiabicdaWaqabaGccqGGPaqkcqGGUaGlaaa@7315@

For time-independent Δ*F*_**ab**_, the stationary solution *φ *(*x*_0_) = lim_*t*→∞ _P
 MathType@MTEF@5@5@+=feaafiart1ev1aaatCvAUfKttLearuWrP9MDH5MBPbIqV92AaeXatLxBI9gBaebbnrfifHhDYfgasaacH8akY=wiFfYdH8Gipec8Eeeu0xXdbba9frFj0=OqFfea0dXdd9vqai=hGuQ8kuc9pgc9s8qqaq=dirpe0xb9q8qiLsFr0=vr0=vr0dc8meaabaqaciaacaGaaeqabaqabeGadaaakeaacqqGqbauaaa@2DD3@(*x *= 1, *t*|*x*_0_, *t*_0_) has the form [28,30]

φ(x0,ΔFab,N)=1−exp⁡(−2NΔFabx0)1−exp⁡(−2NΔFab),
 MathType@MTEF@5@5@+=feaafiart1ev1aaatCvAUfKttLearuWrP9MDH5MBPbIqV92AaeXatLxBI9gBaebbnrfifHhDYfgasaacH8akY=wiFfYdH8Gipec8Eeeu0xXdbba9frFj0=OqFfea0dXdd9vqai=hGuQ8kuc9pgc9s8qqaq=dirpe0xb9q8qiLsFr0=vr0=vr0dc8meaabaqaciaacaGaaeqabaqabeGadaaakeaaiiGacqWFgpGzcqGGOaakcqWG4baEdaWgaaWcbaGaeGimaadabeaakiabcYcaSiabgs5aejabdAeagnaaBaaaleaaieqacqGFHbqycqGFIbGyaeqaaOGaeiilaWIaemOta4KaeiykaKIaeyypa0ZaaSaaaeaacqaIXaqmcqGHsislcyGGLbqzcqGG4baEcqGGWbaCcqGGOaakcqGHsislcqaIYaGmcqWGobGtcqGHuoarcqWGgbGrdaWgaaWcbaGae4xyaeMae4NyaigabeaakiabdIha4naaBaaaleaacqaIWaamaeqaaOGaeiykaKcabaGaeGymaeJaeyOeI0IagiyzauMaeiiEaGNaeiiCaaNaeiikaGIaeyOeI0IaeGOmaiJaemOta4KaeyiLdqKaemOray0aaSbaaSqaaiab+fgaHjab+jgaIbqabaGccqGGPaqkaaGaeiilaWcaaa@5F8B@

which for near-neutral evolution (*NΔF*_**ab **_≪ 1) reduces to

*φ*(*x*_0_, 0, *N*) = *x*_0 _+ *NΔF*_**ab **_*x*_0_(1 - *x*_0_) + ....

The characteristic time *τ*_*s *_of the stochastic dynamics interpolates between the diffusive scale *N *and the deterministic scale: *τ*_*s *_≈ min(*N*, *τ*_*d*_). It determines the typical time of the evolution process up to fixation, shown shaded in fig. 6(b).

Hence, the stochastic population dynamics depends no longer only on the fitness difference of the genotypes as in the deterministic case, but also on the initial state of the population and the the population size. Yet, our evolutionary picture is still incomplete. Population states with coexisting genotypes enter the dynamics as initial conditions, but since mutations are neglected, the model does not explain how this coexistence is generated and maintained.

### Mutation processes and evolutionary equilibria

At the level of an individual, mutations are rare stochastic genotype changes **a **→ **b**, which take place with rates *μ*_**a**→**b**_, often coupled to the reproduction process. (These rates are all of the same order of magnitude, in estimates we therefore omit the indices.) We include mutations into the population dynamics (34) by their systematic effect on the genotype subpopulations,

ddtNa(t)=Fa(t)Na(t)+∑b[μb→aNb(t)−μa→bNa(t)]+χa(t),
 MathType@MTEF@5@5@+=feaafiart1ev1aaatCvAUfKttLearuWrP9MDH5MBPbIqV92AaeXatLxBI9gBaebbnrfifHhDYfgasaacH8akY=wiFfYdH8Gipec8Eeeu0xXdbba9frFj0=OqFfea0dXdd9vqai=hGuQ8kuc9pgc9s8qqaq=dirpe0xb9q8qiLsFr0=vr0=vr0dc8meaabaqaciaacaGaaeqabaqabeGadaaakeaadaWcaaqaaiabbsgaKbqaaiabbsgaKjabdsha0baacqWGobGtdaWgaaWcbaacbeGae8xyaegabeaakiabcIcaOiabdsha0jabcMcaPiabg2da9iabdAeagnaaBaaaleaacqWFHbqyaeqaaOGaeiikaGIaemiDaqNaeiykaKIaemOta40aaSbaaSqaaiab=fgaHbqabaGccqGGOaakcqWG0baDcqGGPaqkcqGHRaWkdaaeqbqaaiabcUfaBHGaciab+X7aTnaaBaaaleaacqWFIbGycqGHsgIRcqWFHbqyaeqaaOGaemOta40aaSbaaSqaaiab=jgaIbqabaGccqGGOaakcqWG0baDcqGGPaqkcqGHsislcqGF8oqBdaWgaaWcbaGae8xyaeMaeyOKH4Qae8Nyaigabeaakiabd6eaonaaBaaaleaacqWFHbqyaeqaaOGaeiikaGIaemiDaqNaeiykaKIaeiyxa0faleaacqWFIbGyaeqaniabggHiLdGccqGHRaWkcqGFhpWydaWgaaWcbaGae8xyaegabeaakiabcIcaOiabdsha0jabcMcaPiabcYcaSaaa@6B50@

while their stochastic effect (whose variance is of order *Nμ*) is neglected since it is small against the reproductive sampling noise *χ*_**a**_(*t*). In the case of two different genotypes, this dynamics can again be projected onto the variable *x*,

ddtx(t)=ΔFab(t)x(t)[1−x(t)]+μa→b[1−x(t)]−μb→ax(t)+χx(t),
 MathType@MTEF@5@5@+=feaafiart1ev1aaatCvAUfKttLearuWrP9MDH5MBPbIqV92AaeXatLxBI9gBaebbnrfifHhDYfgasaacH8akY=wiFfYdH8Gipec8Eeeu0xXdbba9frFj0=OqFfea0dXdd9vqai=hGuQ8kuc9pgc9s8qqaq=dirpe0xb9q8qiLsFr0=vr0=vr0dc8meaabaqaciaacaGaaeqabaqabeGadaaakeaadaWcaaqaaGqaaiab=rgaKbqaaiab=rgaKjabdsha0baacqWG4baEcqGGOaakcqWG0baDcqGGPaqkcqGH9aqpcqGHuoarcqWGgbGrdaWgaaWcbaacbeGae4xyaeMae4NyaigabeaakiabcIcaOiabdsha0jabcMcaPiabdIha4jabcIcaOiabdsha0jabcMcaPiabcUfaBjabigdaXiabgkHiTiabdIha4jabcIcaOiabdsha0jabcMcaPiabc2faDjabgUcaRGGaciab9X7aTnaaBaaaleaacqGFHbqycqGHsgIRcqGFIbGyaeqaaOGaei4waSLaeGymaeJaeyOeI0IaemiEaGNaeiikaGIaemiDaqNaeiykaKIaeiyxa0LaeyOeI0Iae0hVd02aaSbaaSqaaiab+jgaIjabgkziUkab+fgaHbqabaGccqWG4baEcqGGOaakcqWG0baDcqGGPaqkcqGHRaWkcqqFhpWydaWgaaWcbaGaemiEaGhabeaakiabcIcaOiabdsha0jabcMcaPiabcYcaSaaa@70E8@

which leads to the Fokker-Planck equation [31]

∂∂tP(x,t)=1N∂2∂x2x(1−x)P(x,t)−ΔFab(t)∂∂xx(1−x)P(x,t)−μa→b∂∂x(1−x)P(x,t)+μb→a∂∂xxP(x,t).
 MathType@MTEF@5@5@+=feaafiart1ev1aaatCvAUfKttLearuWrP9MDH5MBPbIqV92AaeXatLxBI9gBaebbnrfifHhDYfgasaacH8akY=wiFfYdH8Gipec8Eeeu0xXdbba9frFj0=OqFfea0dXdd9vqai=hGuQ8kuc9pgc9s8qqaq=dirpe0xb9q8qiLsFr0=vr0=vr0dc8meaabaqaciaacaGaaeqabaqabeGadaaakeaafaqaaeGadaaabaWaaSaaaeaacqGHciITaeaacqGHciITcqWG0baDaaGaeeiuaaLaeiikaGIaemiEaGNaeiilaWIaemiDaqNaeiykaKcabaGaeyypa0dabaWaaSaaaeaacqaIXaqmaeaacqWGobGtaaWaaSaaaeaacqGHciITdaahaaWcbeqaaiabikdaYaaaaOqaaiabgkGi2kabdIha4naaCaaaleqabaGaeGOmaidaaaaakiabdIha4jabcIcaOiabigdaXiabgkHiTiabdIha4jabcMcaPiabbcfaqjabcIcaOiabdIha4jabcYcaSiabdsha0jabcMcaPiabgkHiTiabgs5aejabdAeagnaaBaaaleaaieqacqWFHbqycqWFIbGyaeqaaOGaeiikaGIaemiDaqNaeiykaKYaaSaaaeaacqGHciITaeaacqGHciITcqWG4baEaaGaemiEaGNaeiikaGIaeGymaeJaeyOeI0IaemiEaGNaeiykaKIaeeiuaaLaeiikaGIaemiEaGNaeiilaWIaemiDaqNaeiykaKcabaaabaaabaGaeyOeI0ccciGae4hVd02aaSbaaSqaaiab=fgaHjabgkziUkab=jgaIbqabaGcdaWcaaqaaiabgkGi2cqaaiabgkGi2kabdIha4baacqGGOaakcqaIXaqmcqGHsislcqWG4baEcqGGPaqkcqqGqbaucqGGOaakcqWG4baEcqGGSaalcqWG0baDcqGGPaqkcqGHRaWkcqGF8oqBdaWgaaWcbaGae8NyaiMaeyOKH4Qae8xyaegabeaakmaalaaabaGaeyOaIylabaGaeyOaIyRaemiEaGhaaiabdIha4jabbcfaqjabcIcaOiabdIha4jabcYcaSiabdsha0jabcMcaPiabc6caUaaaaaa@956F@

For time-independent Δ*F*_**ab**_, this equation has a single stable stationary state,

P(x)=1Zx−1+Nμa→b(1−x)−1+Nμb→aexp⁡(2NΔFabx)
 MathType@MTEF@5@5@+=feaafiart1ev1aaatCvAUfKttLearuWrP9MDH5MBPbIqV92AaeXatLxBI9gBaebbnrfifHhDYfgasaacH8akY=wiFfYdH8Gipec8Eeeu0xXdbba9frFj0=OqFfea0dXdd9vqai=hGuQ8kuc9pgc9s8qqaq=dirpe0xb9q8qiLsFr0=vr0=vr0dc8meaabaqaciaacaGaaeqabaqabeGadaaakeaacqqGqbaucqGGOaakcqWG4baEcqGGPaqkcqGH9aqpdaWcaaqaaiabigdaXaqaaiabdQfaAbaacqWG4baEdaahaaWcbeqaaiabgkHiTiabigdaXiabgUcaRiabd6eaoHGaciab=X7aTnaaBaaameaaieqacqGFHbqycqGHsgIRcqGFIbGyaeqaaaaakiabcIcaOiabigdaXiabgkHiTiabdIha4jabcMcaPmaaCaaaleqabaGaeyOeI0IaeGymaeJaey4kaSIaemOta4Kae8hVd02aaSbaaWqaaiab+jgaIjabgkziUkab+fgaHbqabaaaaOGagiyzauMaeiiEaGNaeiiCaaNaeiikaGIaeGOmaiJaemOta4KaeyiLdqKaemOray0aaSbaaSqaaiab+fgaHjab+jgaIbqabaGccqWG4baEcqGGPaqkaaa@5E76@

with a normalization constant *Z *that can be expressed in terms of Bessel and Gamma functions [32].

### Substitution dynamics

Here we are interested in the stochastic evolution (45) and its equilibrium state (46) for *Nμ *≪ 1, which is the relevant dynamical regime for nuclear DNA in eukaryotes and in most prokaryotes (but not in viral systems). In this regime, the mutation term in (45) is small against the diffusion term except for values of *x *close to the boundaries 0 or 1. In this region, the continuum approximation of eq. (45) is no longer valid, and (46) has to be replaced by a stationary solution P
 MathType@MTEF@5@5@+=feaafiart1ev1aaatCvAUfKttLearuWrP9MDH5MBPbIqV92AaeXatLxBI9gBaebbnrfifHhDYfgasaacH8akY=wiFfYdH8Gipec8Eeeu0xXdbba9frFj0=OqFfea0dXdd9vqai=hGuQ8kuc9pgc9s8qqaq=dirpe0xb9q8qiLsFr0=vr0=vr0dc8meaabaqaciaacaGaaeqabaqabeGadaaakeaacqqGqbauaaa@2DD3@_*d*_(*N*_**a**_) of the underlying discrete evolution model, which gives the probability that the population contains *N*_**a **_individuals of genotype **a **(with *N*_**a **_= *N *- *N*_b _= 0, 1,..., *N*). The discrete solution is easily shown to have the singularity Pd(0)≃(Nμa→b)−1Pd(1)
 MathType@MTEF@5@5@+=feaafiart1ev1aaatCvAUfKttLearuWrP9MDH5MBPbIqV92AaeXatLxBI9gBaebbnrfifHhDYfgasaacH8akY=wiFfYdH8Gipec8Eeeu0xXdbba9frFj0=OqFfea0dXdd9vqai=hGuQ8kuc9pgc9s8qqaq=dirpe0xb9q8qiLsFr0=vr0=vr0dc8meaabaqaciaacaGaaeqabaqabeGadaaakeaacqqGqbaudaWgaaWcbaGaemizaqgabeaakiabcIcaOiabicdaWiabcMcaPiabloKi7iabcIcaOiabd6eaoHGaciab=X7aTnaaBaaaleaaieqacqGFHbqycqGHsgIRcqGFIbGyaeqaaOGaeiykaKYaaWbaaSqabeaacqGHsislcqaIXaqmaaGccqWGqbaudaWgaaWcbaGaemizaqgabeaakiabcIcaOiabigdaXiabcMcaPaaa@43E0@. This singularity is correctly captured if we use the approximation Pd(Na)≃∫Na/N(Na+1)/NdxP(x)
 MathType@MTEF@5@5@+=feaafiart1ev1aaatCvAUfKttLearuWrP9MDH5MBPbIqV92AaeXatLxBI9gBaebbnrfifHhDYfgasaacH8akY=wiFfYdH8Gipec8Eeeu0xXdbba9frFj0=OqFfea0dXdd9vqai=hGuQ8kuc9pgc9s8qqaq=dirpe0xb9q8qiLsFr0=vr0=vr0dc8meaabaqaciaacaGaaeqabaqabeGadaaakeaacqWGqbaudaWgaaWcbaGaemizaqgabeaakiabcIcaOiabd6eaonaaBaaaleaaieqacqWFHbqyaeqaaOGaeiykaKIaeS4qISZaa8qmaeaacqWGKbazcqWG4baEcqqGqbaucqGGOaakcqWG4baEcqGGPaqkaSqaaiabd6eaonaaBaaameaacqWFHbqyaeqaaSGaei4la8IaemOta4eabaGaeiikaGIaemOta40aaSbaaWqaaiab=fgaHbqabaWccqGHRaWkcqaIXaqmcqGGPaqkcqGGVaWlcqWGobGta0Gaey4kIipaaaa@4B12@ for all *N*_**a **_(except at the other boundary, where there is a similar singularity Pd(N)≃(Nμb→a)−1Pd(N−1))
 MathType@MTEF@5@5@+=feaafiart1ev1aaatCvAUfKttLearuWrP9MDH5MBPbIqV92AaeXatLxBI9gBaebbnrfifHhDYfgasaacH8akY=wiFfYdH8Gipec8Eeeu0xXdbba9frFj0=OqFfea0dXdd9vqai=hGuQ8kuc9pgc9s8qqaq=dirpe0xb9q8qiLsFr0=vr0=vr0dc8meaabaqaciaacaGaaeqabaqabeGadaaakeaacqqGqbaudaWgaaWcbaGaemizaqgabeaakiabcIcaOiabd6eaojabcMcaPiabloKi7iabcIcaOiabd6eaoHGaciab=X7aTnaaBaaaleaaieqacqGFIbGycqGHsgIRcqGFHbqyaeqaaOGaeiykaKYaaWbaaSqabeaacqGHsislcqaIXaqmaaGccqWGqbaudaWgaaWcbaGaemizaqgabeaakiabcIcaOiabd6eaojabgkHiTiabigdaXiabcMcaPiabcMcaPaaa@4703@[33].

From this solution, we read off the following characteristics of the evolutionary dynamics at equilibrium, which are illustrated by the trajectory of fig. 6(c)[32]:

(a) For sufficiently small values of *μ*, the population remains monomorphic for most of the time. Using the shorthands *Q*(**a**) = P
 MathType@MTEF@5@5@+=feaafiart1ev1aaatCvAUfKttLearuWrP9MDH5MBPbIqV92AaeXatLxBI9gBaebbnrfifHhDYfgasaacH8akY=wiFfYdH8Gipec8Eeeu0xXdbba9frFj0=OqFfea0dXdd9vqai=hGuQ8kuc9pgc9s8qqaq=dirpe0xb9q8qiLsFr0=vr0=vr0dc8meaabaqaciaacaGaaeqabaqabeGadaaakeaacqqGqbauaaa@2DD3@_*d*_(*N*_**a **_= 0) and *Q*(**b**) = P
 MathType@MTEF@5@5@+=feaafiart1ev1aaatCvAUfKttLearuWrP9MDH5MBPbIqV92AaeXatLxBI9gBaebbnrfifHhDYfgasaacH8akY=wiFfYdH8Gipec8Eeeu0xXdbba9frFj0=OqFfea0dXdd9vqai=hGuQ8kuc9pgc9s8qqaq=dirpe0xb9q8qiLsFr0=vr0=vr0dc8meaabaqaciaacaGaaeqabaqabeGadaaakeaacqqGqbauaaa@2DD3@_*d*_(*N*_**a **_= *N*), we have

*Q*(**a**) + *Q*(**b**) = 1 - *O*(*μN *log *N*).

(b) The ratio of probabilities for the two monomorphic population states is given by the ratio of "forward" and "backward" mutation rate, the fitness difference, and the effective population size:

Q(b)Q(a)=μa→bμb→aexp⁡(2NΔFab)+O(Nμ).
 MathType@MTEF@5@5@+=feaafiart1ev1aaatCvAUfKttLearuWrP9MDH5MBPbIqV92AaeXatLxBI9gBaebbnrfifHhDYfgasaacH8akY=wiFfYdH8Gipec8Eeeu0xXdbba9frFj0=OqFfea0dXdd9vqai=hGuQ8kuc9pgc9s8qqaq=dirpe0xb9q8qiLsFr0=vr0=vr0dc8meaabaqaciaacaGaaeqabaqabeGadaaakeaadaWcaaqaaiabdgfarjabcIcaOGqabiab=jgaIjabcMcaPaqaaiabdgfarjabcIcaOiab=fgaHjabcMcaPaaacqGH9aqpdaWcaaqaaGGaciab+X7aTnaaBaaaleaacqWFHbqycqGHsgIRcqWFIbGyaeqaaaGcbaGae4hVd02aaSbaaSqaaiab=jgaIjabgkziUkab=fgaHbqabaaaaOGagiyzauMaeiiEaGNaeiiCaaNaeiikaGIaeGOmaiJaemOta4KaeyiLdqKaemOray0aaSbaaSqaaiab=fgaHjab=jgaIbqabaGccqGGPaqkcqGHRaWkcqWGpbWtcqGGOaakcqWGobGtdaWgaaWcbaGae4hVd0gabeaakiabcMcaPiabc6caUaaa@57E1@

(c) The monomorphic population states *x *= 0 and *x *= 1 are unstable due to mutations even at arbitrarily small values of *μ*, which cause occasional transitions of the entire population from genotype **a **to **b**, and vice versa. These so-called *substitutions *are marked by dashed lines in fig. 6(c). The substitution rate *u*_**a**→**b **_can be evaluated as the product of creating a single mutant of genotype **b **in an initially monomorphic **a **population, Nμa→b
 MathType@MTEF@5@5@+=feaafiart1ev1aaatCvAUfKttLearuWrP9MDH5MBPbIqV92AaeXatLxBI9gBaebbnrfifHhDYfgasaacH8akY=wiFfYdH8Gipec8Eeeu0xXdbba9frFj0=OqFfea0dXdd9vqai=hGuQ8kuc9pgc9s8qqaq=dirpe0xb9q8qiLsFr0=vr0=vr0dc8meaabaqaciaacaGaaeqabaqabeGadaaakeaacqWGobGtiiGacqWF8oqBdaWgaaWcbaacbeGae4xyaeMaeyOKH4Qae4Nyaigabeaaaaa@343F@, and its probability of fixation, *φ *(*x*_0 _= 1/*N*, Δ*F*_**ab**_, *N*). The time between initial mutation and fixation (shown by grey shading in fig. 6(c)) is still of order *τ*_*s *_and thus much shorter than the time scale 1/*μ*, on which mutation effects become important. Hence, the fixation probability *φ *is given to leading order by (41), which has been derived for *μ *= 0. Together we have [28,30]

ua→b=Nμa→b1−exp⁡(−2ΔFab)1−exp⁡(−2NΔFab).
 MathType@MTEF@5@5@+=feaafiart1ev1aaatCvAUfKttLearuWrP9MDH5MBPbIqV92AaeXatLxBI9gBaebbnrfifHhDYfgasaacH8akY=wiFfYdH8Gipec8Eeeu0xXdbba9frFj0=OqFfea0dXdd9vqai=hGuQ8kuc9pgc9s8qqaq=dirpe0xb9q8qiLsFr0=vr0=vr0dc8meaabaqaciaacaGaaeqabaqabeGadaaakeaacqWG1bqDdaWgaaWcbaacbeGae8xyaeMaeyOKH4Qae8Nyaigabeaaiiaakiab+1da9iabd6eaoHGaciab9X7aTnaaBaaaleaacqWFHbqycqGHsgIRcqWFIbGyaeqaaOWaaSaaaeaacqaIXaqmcqGHsislcyGGLbqzcqGG4baEcqGGWbaCcqGGOaakcqGHsislcqaIYaGmcqGHuoarcqWGgbGrdaWgaaWcbaGae8xyaeMae8NyaigabeaakiabcMcaPaqaaiabigdaXiabgkHiTiGbcwgaLjabcIha4jabcchaWjabcIcaOiabgkHiTiabikdaYiabd6eaojabgs5aejabdAeagnaaBaaaleaacqWFHbqycqWFIbGyaeqaaOGaeiykaKcaaiabc6caUaaa@5B58@

**Figure 7:**
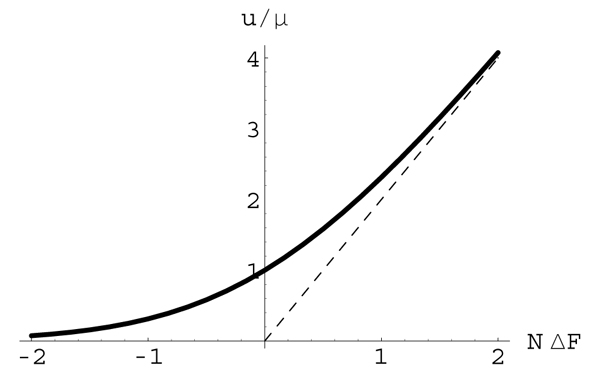
**Substitution rate in a population versus mutation rate in an individual**. The ratio of these rates, *u*_**a**→**b**_/*μ*_**a**→**b**_, depends on the product *N*Δ*F*_**ab **_of effective population size and fitness difference between the genotypes (in the relevant regime *N *≫ 1, Δ*F*_**ab **_≪ 1, *N*Δ*F*_**ab **_finite). The substitution rate *u*_**a**→**b **_is equal to *μ*_**ab **_for neutral mutations (Δ*F*_**ab **_= 0), reduced for deleterious mutations (Δ*F*_**ab **_< 0), and enhanced for advantageous mutations (Δ*F*_**ab **_> 0).

Hence, the substitution rate *u*_**a**→**b **_is enhanced over *μ*_**a**→**b **_for Δ*F*_**ab **_> 0 and suppressed for Δ*F*_**ab **_< 0, as shown in Fig. 7. For weak selection (*N*|Δ*F*_**ab**_| ≪ 1), eq. (49) becomes

*u*_**a**→**b **_= *μ*_**a**→**b **_(1 + *NΔF*_**ab **_+ ...).

This reproduces Kimura's famous original result: for neutral evolution, the substitution rate equals the mutation rate in an individual, independently of the population size. For this reason, the rates *μ*_**a**→**b **_are referred to as neutral mutation rates. For strong selection (*N*|Δ*F*_**ab**_| ≫ 1 ≫ |Δ*F*_**ab**_|), eq. (49) takes the asymptotic forms

ua→b=μa→b{2N|ΔFab|exp⁡(2NΔFab)(2NΔFab≪1),2NΔFab(2NΔFab≫1).
 MathType@MTEF@5@5@+=feaafiart1ev1aaatCvAUfKttLearuWrP9MDH5MBPbIqV92AaeXatLxBI9gBaebbnrfifHhDYfgasaacH8akY=wiFfYdH8Gipec8Eeeu0xXdbba9frFj0=OqFfea0dXdd9vqai=hGuQ8kuc9pgc9s8qqaq=dirpe0xb9q8qiLsFr0=vr0=vr0dc8meaabaqaciaacaGaaeqabaqabeGadaaakeaacqWG1bqDdaWgaaWcbaacbeGae8xyaeMaeyOKH4Qae8Nyaigabeaakiabg2da9GGaciab+X7aTnaaBaaaleaacqWFHbqycqGHsgIRcqWFIbGyaeqaaOWaaiqaaeaafaqaaeGacaaabaGaeGOmaiJaemOta4KaeiiFaWNaeyiLdqKaemOray0aaSbaaSqaaiab=fgaHjab=jgaIbqabaGccqGG8baFcyGGLbqzcqGG4baEcqGGWbaCcqGGOaakcqaIYaGmcqWGobGtcqGHuoarcqWGgbGrdaWgaaWcbaGae8xyaeMae8NyaigabeaakiabcMcaPaqaaiabcIcaOiabikdaYiabd6eaojabgs5aejabdAeagnaaBaaaleaacqWFHbqycqWFIbGyaeqaaOGaeSOAI0JaeGymaeJaeiykaKIaeiilaWcabaGaeGOmaiJaemOta4KaeyiLdqKaemOray0aaSbaaSqaaiab=fgaHjab=jgaIbqabaaakeaacqGGOaakcqaIYaGmcqWGobGtcqGHuoarcqWGgbGrdaWgaaWcbaGae8xyaeMae8NyaigabeaakiablUMi=iabigdaXiabcMcaPiabc6caUaaaaiaawUhaaaaa@72DC@

The backward substitution rate *u*_**b**→**a **_is given by a formula similar to (49) with Δ*F*_**ba **_= -Δ*F*_**ab**_. Forward and backward substitution rate have the simple ratio

ua→bub→a=μa→bμb→aexp⁡(2NΔFab)
 MathType@MTEF@5@5@+=feaafiart1ev1aaatCvAUfKttLearuWrP9MDH5MBPbIqV92AaeXatLxBI9gBaebbnrfifHhDYfgasaacH8akY=wiFfYdH8Gipec8Eeeu0xXdbba9frFj0=OqFfea0dXdd9vqai=hGuQ8kuc9pgc9s8qqaq=dirpe0xb9q8qiLsFr0=vr0=vr0dc8meaabaqaciaacaGaaeqabaqabeGadaaakeaadaWcaaqaaiabdwha1naaBaaaleaaieqacqWFHbqycqGHsgIRcqWFIbGyaeqaaaGcbaGaemyDau3aaSbaaSqaaiab=jgaIjabgkziUkab=fgaHbqabaaaaOGaeyypa0ZaaSaaaeaaiiGacqGF8oqBdaWgaaWcbaGae8xyaeMaeyOKH4Qae8NyaigabeaaaOqaaiab+X7aTnaaBaaaleaacqWFIbGycqGHsgIRcqWFHbqyaeqaaaaakiGbcwgaLjabcIha4jabcchaWjabcIcaOiabikdaYiabd6eaojabgs5aejabdAeagnaaBaaaleaacqWFHbqycqWFIbGyaeqaaOGaeiykaKcaaa@5439@

for *N *≫ 1. Comparing with (48), we obtain the consistency condition

ua→bub→a=Q(b)Q(a).
 MathType@MTEF@5@5@+=feaafiart1ev1aaatCvAUfKttLearuWrP9MDH5MBPbIqV92AaeXatLxBI9gBaebbnrfifHhDYfgasaacH8akY=wiFfYdH8Gipec8Eeeu0xXdbba9frFj0=OqFfea0dXdd9vqai=hGuQ8kuc9pgc9s8qqaq=dirpe0xb9q8qiLsFr0=vr0=vr0dc8meaabaqaciaacaGaaeqabaqabeGadaaakeaadaWcaaqaaiabdwha1naaBaaaleaaieqacqWFHbqycqGHsgIRcqWFIbGyaeqaaaGcbaGaemyDau3aaSbaaSqaaiab=jgaIjabgkziUkab=fgaHbqabaaaaOGaeyypa0ZaaSaaaeaacqWGrbqucqGGOaakcqWFIbGycqGGPaqkaeaacqWGrbqucqGGOaakcqWFHbqycqGGPaqkaaGaeiOla4caaa@4356@

Hence, for sufficiently small mutation rates (*μN *log *N *≪ 1), a simple picture emerges: The evolution of a population can be described as a sequence of transitions between monomorphic genotype states (substitutions). The substitution rate *u *is determined by the corresponding mutation rate in an individual, the fitness difference between the genotypes, and the effective population size.

### Neutral dynamics in sequence space, sequence entropy

This evolutionary picture can be generalized to multiple genotypes, for example, the 4^ℓ ^dimensional sequence space of genomic loci **a **= (*a*_1_,..., *a*_ℓ_). Transitions between different sequence states are point mutations **a **→ **b**, which change exactly one nucleotide. (We neglect here insertion and deletion processes, which change the length of the sequence). We first discuss neutral evolution, where the substitution rate *u*_**a**→**b **_equals the mutation rate in an individual, *μ*_**a**→**b**_, for all elementary transitions **a **→ **b**. Bona fide neutral mutation rates can be inferred from DNA sequence alignments of sufficiently close species, recent insights have also come from studying repeat elements.

We assume the neutral dynamics has an equilibrium distribution *P*_0_(**a**) which obeys *detailed balance*, i.e., the relation

μa→bμb→a=P0(b)P0(a)
 MathType@MTEF@5@5@+=feaafiart1ev1aaatCvAUfKttLearuWrP9MDH5MBPbIqV92AaeXatLxBI9gBaebbnrfifHhDYfgasaacH8akY=wiFfYdH8Gipec8Eeeu0xXdbba9frFj0=OqFfea0dXdd9vqai=hGuQ8kuc9pgc9s8qqaq=dirpe0xb9q8qiLsFr0=vr0=vr0dc8meaabaqaciaacaGaaeqabaqabeGadaaakeaadaWcaaqaaGGaciab=X7aTnaaBaaaleaaieqacqGFHbqycqGHsgIRcqGFIbGyaeqaaaGcbaGae8hVd02aaSbaaSqaaiab+jgaIjabgkziUkab+fgaHbqabaaaaOGaeyypa0ZaaSaaaeaacqWGqbaudaWgaaWcbaGaeGimaadabeaakiabcIcaOiab+jgaIjabcMcaPaqaaiabdcfaqnaaBaaaleaacqaIWaamaeqaaOGaeiikaGIae4xyaeMaeiykaKcaaaaa@4538@

holds for each pair of sequence states linked by an elementary transition process **a **→ **b**. This says that the probability current at equilibrium, *μ*_**a**→**b**_*P*_0_(**a**) - *μ*_**b**→**a**_*P*_0_(**b**), vanishes for each elementary transition. Clearly, any distribution *P*_0_(**a**) satisfying the conditions (54) is stationary under the dynamics with rates *μ*_**a**→**b**_, but not every such dynamics has a stationary distribution which satisfies (54) (the simplest counterexample involving three states and a circular probability current **a **→ **b **→ **c **at stationarity).

However, as will be verified below, detailed balance is a good approximation for the genomic substitution dynamics at least in prokaryotes. (There are known violations at CpG islands in eukaryotes [34]). In the simplest type of models, every nucleotide *a *mutates independently of all other positions with uniform rates *μ*_*a*→*b *_(i.e., *μ*_**a**→**b **_= *μ*_*a*→*b *_for any two sequences **a **= (..., *a *,...) and **b **= (..., *b*, ...) differing by exactly one nucleotide). This produces a factorized equilibrium distribution *P*_0_(**a**) of the form (15).

We can project the equilibrium distribution onto a measurable quantity as independent variable. For binding site sequences, a convenient choice is the binding energy *E*, and the projected distribution *P*_0_(*E*) has the form (16). Hence we can define the *sequence entropy *[35]

*S*_0_(*E*) = log *P*_0_(*E*),

which counts the log density of sequence states **a **at energy *E*, weighed by the distribution *P*_0_(**a**).

### Dynamics under selection, the score-fitness relation

The dynamics of substitutions can be studied in the same way for evolution under selection, which is specified at the level of genotypes by an arbitrary fitness function *F*(**a**) [18,36]. This generalizes the results of [37] for a model with selection acting independently at different nucleotide positions, i.e., F(a)=∑i=1ℓfi(ai)
 MathType@MTEF@5@5@+=feaafiart1ev1aaatCvAUfKttLearuWrP9MDH5MBPbIqV92AaeXatLxBI9gBaebbnrfifHhDYfgasaacH8akY=wiFfYdH8Gipec8Eeeu0xXdbba9frFj0=OqFfea0dXdd9vqai=hGuQ8kuc9pgc9s8qqaq=dirpe0xb9q8qiLsFr0=vr0=vr0dc8meaabaqaciaacaGaaeqabaqabeGadaaakeaacqWGgbGrcqGGOaakieqacqWFHbqycqGGPaqkcqGH9aqpdaaeWaqaaiabdAgaMnaaBaaaleaacqWGPbqAaeqaaOGaeiikaGIaemyyae2aaSbaaSqaaiabdMgaPbqabaGccqGGPaqkaSqaaiabdMgaPjabg2da9iabigdaXaqaaiabloriSbqdcqGHris5aaaa@3FC2@. For each elementary transition **a **→ **b**, the substitution rate *u*_**a**→**b **_is determined by the neutral rate *μ*_**a**→**b**_, the fitness difference Δ*F*_**ab**_, and the effective population size *N *according to (49). Given the detailed balance (54) of neutral evolution and the relation (52) between forward and backward rates, it then follows immediately that the evolutionary dynamics under selection also obeys detailed balance, as given by (53) with an equilibrium distribution *Q*(**a**) of the form (48). Thus we have [18,36]:

*The equilibrium distribution Q*(**a**) *of fixed genotypes generated by a substitution dynamics *(49) *with fitness function F*(**a**) *is related to its neutral counterpart P*_0_(**a**) *by*

*Q*(**a**) = *P*_0_(**a**) exp [2*NF *(**a**) + const.],

*with the constant given by normalization*.

We can project eq. (56) onto the fitness as independent variable. Defining the distribution *Q*(*F*) ≡ *∑*_**a **_*Q*(**a**)*δ *(*F *(**a**) - *F*), similarly *P*_0_(*F*), and the sequence entropy *S*_0_(*F*) = log *P*_0_(*F*), the projected identity takes the form

*Q*(*F*) = exp [2*NF *+ *S*_0_(*F*) + const.]

For binding site sequences, we have a similar projection on the binding energy, *Q*(*E*) = exp [2*NF*(*E*) + *S*_0_(*E*) + const.], since all genotypes with the same "phenotype" *E *have the same fitness, i.e., the same score *S*. The projected identities express the equilibrium distribution under selection in terms of fitness and sequence entropy, reflecting the balance between stochasticity (genetic drift) and selection [18]. For strong selection, the exponent 2*NF *- *S*_0 _is dominated by the fitness term, and *Q*(*F*) takes appreciable values only at points of near-maximal fitness, i.e., where *F*_*max *_- *F *≲ 1/2*N*. For moderate selection, there is a nontrivial balance between both terms, and for weak selection, the *Q *distribution can be approximated by its neutral counterpart *P*_0 _= exp(*S*_0_). Clearly, the roles of fitness and sequence entropy are formally analogous to those of energy and entropy in statistical physics of thermodynamic systems, if 2*N *is identified with the inverse temperature 1/*k*_*B*_*T *. Some consequences of this analogy are discussed in ref. [38].

The dynamics of substitutions establishes a rather general evolutionary grounding of genome statistics, if we identify the equilibrium distributions *P*_0_(**a**) and *Q*(**a**) with the genomic distributions discussed in the previous section, as already anticipated by our notation. Comparing eqs. (56) and (17) gives a relation between fitness and score [16,18]:

*The log-likelihood score S*(**a**) = log [*Q*(**a**)/*P*_0_(**a**)] *equals the fitness function multiplied by twice the effective population size up to a constant*,

*S*(**a**) = 2*NF*(**a**) + const..

This relation allows us to use sequence data of a given genome to infer quantitative patterns of its evolution. We now discuss specific consequences for the evolution of regulatory DNA; an application to protein evolution can be found in ref. [37].

### Measuring selection for binding sites

We first give a precise definition of functionality for regulatory (and other) elements: A binding *locus *is functional if the genotype at that locus is under selection (for binding of the corresponding factor). Nonfunctional loci have evolutionarily neutral genotypes. This definition asks whether binding at a given locus makes a difference to the organism or not. It is weaker than that of a functional *binding site*, which is a functional locus with a sequence **a **that is likely to actually bind the factor. A functional locus can lose its binding sequence due to deleterious mutations, leading to suboptimal fitness of the organism. Conversely, a nonfunctional locus can have by chance a sequence which does bind the factor: this is a spurious binding site without consequences for the organism. To measure the selection on functional sites *in silico*, we apply the identity (58) to the genomic distributions *P*_0_(**a**) and *Q*(**a**). (Assuming equilibrium for most loci seems to be justified for our example of CRP binding sites in *E. coli *since we find very similar distributions in the distant bacterial species *Salmonella typhimurium*, and the factor protein itself is highly conserved between these species.) After projection onto the energy, the fitness landscape 2*NF*(*E*) for CRP binding sites is thus given by fig. 4(b)[16]. The fitness is constant in the no-binding region (*E *≳ *E*_*s *_≈ 13) since the evolution is always neutral in that region. This constant is set to 0 in our normalization, i.e., *F*(*E*) measures the fitness gain of functional sites due to factor binding. Loci with strong binding are also under strong selection, with effective fitness values 2*NF *of order 10. Genetic drift counteracts selection, producing also loci with weaker binding and reduced effective fitness. This fitness "landscape" is thus qualitatively of the form predicted from the underlying biophysics [18,25]. Of course, it should be kept in mind that this landscape results from averaging over a family of binding sites, which may have a spectrum of individual selection coefficients and selected binding strengths.

### Nucleotide frequency correlations

A further consequence of (57) is the generic occurence of nucleotide frequency correlations within functional loci [18]. If the fitness function *F*(**a**) is not additive in the nucleotide positions, nucleotide frequencies are correlated in selected genotypes even if they are independent under neutral evolution. This happens quite generically since selection acts on the entire genotype **a **as a functional unit and not on its single nucleotides. For binding sites, fitness effects follow from the expression level of the regulated gene, which depends on the sequence **a **via the binding probability of the corresponding transcription factor. While the binding energy is often approximately additive in the nucleotide positions as given by (1), the binding probability (10) is a strongly nonlinear function of the energy. This introduces correlations between nucleotide frequencies at *any two *positions within functional loci, preventing factorization of the distribution *Q*(**a**).

### Stationary evolution of binding sites

**Figure 8:**
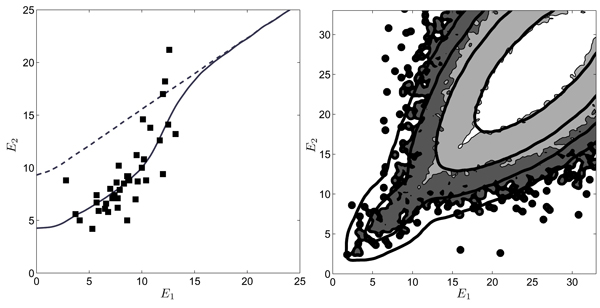
**Evolution of binding sites**. (a) Binding energy pairs (*E*_1_, *E*_2_) for 32 experimentally verified CRP binding sites in *E. coli *from the DPInteract database [57] and their aligned orthologs in *S. typhimurium *(dots). Conditional expectation value for the binding energy in *S. typhimurium *under neutral evolution, ⟨*G*_0_(*E*_2_|*E*_1_)⟩ (dashed line), and under selection, ⟨*G*_*f*_(*E*_2_|*E*_1_)⟩ (solid line). (b) Distribution of energy pair counts *W*_dat_(*E*_1_, *E*_2_) (filled contours), compared to the distribution *W*(*E*_1_, *E*_2_) given by the Bayesian model (62). The symmetry of these distributions under exchange of *E*_1 _and *E*_2 _reflects detailed balance of the substitution dynamics. From [16,39].

Functional loci with a substantial level of selection (as found for the CRP binding sites in *E. coli*) evolve in a way quite different from background sequence. This is quantified in fig. 8(a), which shows pairs of binding energies (*E*_1_, *E*_2_) for experimentally verified CRP binding sites in *E. coli *and the corresponding sites regulating orthologous genes in *S. typhimurium *[16,27]. The evolutionary distance *t *between the two species and characteristics of the neutral mutation process can be inferred from alignments of background sequence. The "phenotypic" evolution of CRP binding is quantified by the *energy transition probabilities G*_0_(*E*_2_|*E*_1_) under neutral evolution and *G*_*f *_(*E*_2_|*E*_1_) under stationary selection [16]. These are readily obtained by simulating the substitution dynamics over a time interval *t *for given initial value *E*_1_, both with neutral rates *μ*_**a**→**b **_and with rates *u*_**a**→**b **_given by (49) and the fitness function 2*NF*(*E*) measured in *E. coli*. The resulting conditional expectation values ⟨*G*_0_(*E*_2_|*E*_1_)⟩ and ⟨*G*_*f *_(*E*_2_|*E*_1_)⟩ for the binding energy in *S. typhimurium *are also shown in fig. 8(a). The data conform to the selection model, showing a substantially stronger conservation of binding energy than expected for neutral evolution [16,27,39].

We can now build a probabilistic model for cross-species comparisons [16]. It is based on the joint distributions of energy pairs

*P*_0_(*E*_1_, *E*_2_) = *G*_0_(*E*_2_|*E*_1_) *P*_0_(*E*_1_)

under neutral evolution and

*Q*(*E*_1_, *E*_2_) = *G*_*f*_(*E*_2_|*E*_1_) *Q*(*E*_1_)

under stationary selection, which are determined by the corresponding distributions in one species and the energy transition probabilities. Detailed balance of the substitution dynamics implies

P0(E2)P0(E1)=G0(E2|E1)G0(E1|E2) and Q(E2)Q(E1)=Gf(E2|E1)Gf(E1|E2),
 MathType@MTEF@5@5@+=feaafiart1ev1aaatCvAUfKttLearuWrP9MDH5MBPbIqV92AaeXatLxBI9gBaebbnrfifHhDYfgasaacH8akY=wiFfYdH8Gipec8Eeeu0xXdbba9frFj0=OqFfea0dXdd9vqai=hGuQ8kuc9pgc9s8qqaq=dirpe0xb9q8qiLsFr0=vr0=vr0dc8meaabaqaciaacaGaaeqabaqabeGadaaakeaadaWcaaqaaiabdcfaqnaaBaaaleaacqaIWaamaeqaaOGaeiikaGIaemyrau0aaSbaaSqaaiabikdaYaqabaGccqGGPaqkaeaacqWGqbaudaWgaaWcbaGaeGimaadabeaakiabcIcaOiabdweafnaaBaaaleaacqaIXaqmaeqaaOGaeiykaKcaaiabg2da9maalaaabaGaem4raC0aaSbaaSqaaiabicdaWaqabaGccqGGOaakcqWGfbqrdaWgaaWcbaGaeGOmaidabeaakiabcYha8jabdweafnaaBaaaleaacqaIXaqmaeqaaOGaeiykaKcabaGaem4raC0aaSbaaSqaaiabicdaWaqabaGccqGGOaakcqWGfbqrdaWgaaWcbaGaeGymaedabeaakiabcYha8jabdweafnaaBaaaleaacqaIYaGmaeqaaOGaeiykaKcaaiabbccaGiabbggaHjabb6gaUjabbsgaKjabbccaGmaalaaabaGaemyuaeLaeiikaGIaemyrau0aaSbaaSqaaiabikdaYaqabaGccqGGPaqkaeaacqWGrbqucqGGOaakcqWGfbqrdaWgaaWcbaGaeGymaedabeaakiabcMcaPaaacqGH9aqpdaWcaaqaaiabdEeahnaaBaaaleaacqWGMbGzaeqaaOGaeiikaGIaemyrau0aaSbaaSqaaiabikdaYaqabaGccqGG8baFcqWGfbqrdaWgaaWcbaGaeGymaedabeaakiabcMcaPaqaaiabdEeahnaaBaaaleaacqWGMbGzaeqaaOGaeiikaGIaemyrau0aaSbaaSqaaiabigdaXaqabaGccqGG8baFcqWGfbqrdaWgaaWcbaGaeGOmaidabeaakiabcMcaPaaacqGGSaalaaa@7453@

i.e., the joint distributions *P*_0_(*E*_1_, *E*_2_) and *Q*(*E*_1_, *E*_2_) must be symmetric functions of their arguments. These distributions combine into a model for pairs of aligned loci, which generalizes the single-species model (25) and takes the form

*W*(*E*_1_, *E*_2_) = (1 - *λ*)*P*_0_(*E*_1_, *E*_2_) + *λQ*(*E*_1_, *E*_2_).

(This model can be extended further to include non-stationary selection.) The distribution *W*(*E*_1_, *E*_2_) with a fraction of functionality *λ *= 0.0018 is in excellent agreement with the count distribution *W*_dat_(*E*_1_, *E*_2_) obtained from *E. coli *and *S. typhimurium*, as shown in fig. 8(b). The symmetry of *W*_dat _is thus consistent with the underlying assumption of detailed balance. Analogous Bayesian models can be defined for more than two species related by a phylogeny. This approach has been applied to binding site prediction in bacteria [16]; a related study of several species of funghi has been reported in ref. [40].

### Adaptive evolution of binding sites

What does this picture say about the adaptive evolution of transcriptional regulation in response to a newly arising selection pressure? The evolution from a genotype with marginal binding (*E*(**a**) ≈ *E*_*s*_) to strong binding requires only about three uphill point mutations in the fitness landscape of fig. 4(b), i.e., there is an effective fitness gain 2*NΔF *≈ 3 per mutation. Hence, according to (51), the rate of uphill substitutions per locus is enhanced by a factor 2*NΔF*·*d*(**a**, a*) at least of order 10 over the neutral point mutation rate per nucleotide. At the same time, the downhill rate is strongly suppressed. This shows that the adaptive formation of a binding site from background sequence can indeed be a rapid mode of regulatory evolution, due to the substantial level of selection [18].

However, this mode is only efficient if adaptation can set in immediately after the selection pressure is established. In larger regulatory regions, the exact position of a binding site is often not important. We assume the initial genome contains a set of L˜
 MathType@MTEF@5@5@+=feaafiart1ev1aaatCvAUfKttLearuWrP9MDH5MBPbIqV92AaeXatLxBI9gBaebbnrfifHhDYfgasaacH8akY=wiFfYdH8Gipec8Eeeu0xXdbba9frFj0=OqFfea0dXdd9vqai=hGuQ8kuc9pgc9s8qqaq=dirpe0xb9q8qiLsFr0=vr0=vr0dc8meaabaqaciaacaGaaeqabaqabeGadaaakeaacuWGmbatgaacaaaa@2DDC@*shadow sites*, i.e., positions *r*_1_,..., rL˜
 MathType@MTEF@5@5@+=feaafiart1ev1aaatCvAUfKttLearuWrP9MDH5MBPbIqV92AaeXatLxBI9gBaebbnrfifHhDYfgasaacH8akY=wiFfYdH8Gipec8Eeeu0xXdbba9frFj0=OqFfea0dXdd9vqai=hGuQ8kuc9pgc9s8qqaq=dirpe0xb9q8qiLsFr0=vr0=vr0dc8meaabaqaciaacaGaaeqabaqabeGadaaakeaacqWGYbGCdaWgaaWcbaGafmitaWKbaGaaaeqaaaaa@2F75@ where a given sequence **a **would have the same regulatory effect. If one of these shadow sites has already a genotype with marginal binding, it acts as a "seed" for the onset of adaptation [41]. On the other hand, if all shadow sites of the initial genome have energy *E *> *E*_*s*_, there is typically a substantial waiting time of neutral evolution before one of them reaches the threshold energy *E*_*s*_. Assuming the initial genome to be entirely background sequence, it will contain at least one such seed if ∫E<EsP0(E)dE≳1/L˜
 MathType@MTEF@5@5@+=feaafiart1ev1aaatCvAUfKttLearuWrP9MDH5MBPbIqV92AaeXatLxBI9gBaebbnrfifHhDYfgasaacH8akY=wiFfYdH8Gipec8Eeeu0xXdbba9frFj0=OqFfea0dXdd9vqai=hGuQ8kuc9pgc9s8qqaq=dirpe0xb9q8qiLsFr0=vr0=vr0dc8meaabaqaciaacaGaaeqabaqabeGadaaakeaadaWdraqaceaaOAGaemiuaa1aaSbaaSqaaiabicdaWaqabaGccqGGOaakcqWGfbqrcqGGPaqkcqqGKbazcqWGfbqraSqaaiabdweafjabgYda8iabdweafnaaBaaameaacqWGZbWCaeqaaaWcbeqdcqGHRiI8amrtHrhAL1wy0L2yHndaryqtHrhAL1wy0L2yHndaiqaakiab=nNi0jabigdaXiabc+caViqbdYeamzaaiaaaaa@49A2@, which is a joint condition on L˜
 MathType@MTEF@5@5@+=feaafiart1ev1aaatCvAUfKttLearuWrP9MDH5MBPbIqV92AaeXatLxBI9gBaebbnrfifHhDYfgasaacH8akY=wiFfYdH8Gipec8Eeeu0xXdbba9frFj0=OqFfea0dXdd9vqai=hGuQ8kuc9pgc9s8qqaq=dirpe0xb9q8qiLsFr0=vr0=vr0dc8meaabaqaciaacaGaaeqabaqabeGadaaakeaacuWGmbatgaacaaaa@2DDC@ and the site length ℓ: the shadow regulatory region must be long enough and binding sites must be short enough. The example shows that the evolvability of regulation imposes constraints on genome architecture [18]. Adaptive point substitution may thus be a feasible mode for the formation of a single binding site, but will hardly explain the groups of adjacent sites characteristic of eukaryotic promoters. These may originate from repeat duplication by slippage, which has recently been shown to be an efficient source of sequence innovation in intergenic regions of *Drosophila*.

## Towards a dynamical picture of the genome

The relationship *S *= 2*NF *+ const. between score and fitness is a cornerstone of the theoretical picture developed so far, which links its population genetic, bioinformatic and biophysical arches. It relates a key evolutionary variable with the statistics of genomic frequency counts. The physical binding energy is an appropriate phenotypic variable on which fitness and score depend, because molecular function is determined by binding interactions.

We have discussed this picture for transcription factor binding sites, but it can be applied more generally to functional elements in genomes. It relates the statistics of these elements in one genome with their evolutionary dynamics, which is observed in cross-species comparisons. This dynamics is shaped by selection: The components of functional elements are coupled by a common fitness function; such fitness interactions are called *epistasis*. Hence, *functional correlations lead to evolutionary correlations*. These can be traced in the *Q *distribution over fixed genomes of a functional element. A more detailed statistical analysis using the statistics of polymorphisms within a population is briefly sketched below.

Thus, the picture of the genome as a system with multiple interactions has a fundamental dynamical significance. This is important since it allows us to trace functional modules from evolutionary patterns. We conclude the article with a brief outlook on functional integration of regulatory sequences at various and its dynamical implications.

### Evolutionary interactions between sites

Regulatory function is often determined not by single binding sites, but jointly by a group of sites in the same regulatory region [42]. An important mechanism is *binding cooperativity*, i.e., the formation of a protein complex between two (or more) factors bound to their corresponding DNA sites. The binding energy of this complex has the form *E *= *E*_1 _+ *E*_2 _+ Δ*E*_12_, where *E*_1 _and *E*_2 _are the energies of the factors bound individually and Δ*E*_12 _< 0 is the energy gain due to the protein-protein interaction, which is of the order of a few *k*_*B*_*T *. Cooperative binding has a number of functional effects [1]:

(a) It increases the signal-to-noise ratio for the targeting of regulatory input to a specific gene, which is important in larger eukaryotic genomes, where single spurious binding sites are abundant in background sequence.

(b) It sharpens the response of the binding probability to variations in the factor concentrations around their threshold value. This follows from the thermodynamics of two factors, which is a straightforward generalization of the case of a single factor discussed above.

(c) It implements logical connections between regulatory input signals to a given gene. The simplest example is an AND connection between two factors, where the regulated gene is affected only if both factors are simultaneously present. This happens if the binding energies and factor concentrations are such that individual binding is weak but joint binding is strong. Larger groups of binding sites can encode a whole repertoire of more complicated logical functions [43].

Regulatory modules with several jointly acting binding sites are frequently found in eukaryotes. The functional coupling of sites in a module translates into interactions between these sites in their sequence evolution. The genomic functional element, i.e., the subset of the regulatory region on which selection acts, is the module as a whole. Its fitness *F*(*E*_1_, *E*_2_, Δ*E*_12_,...) is a joint function of the binding energies as the relevant phenotypic variables [18,25]. The evolutionary dynamics under this selection allows for a large number of *compensatory changes*, i.e., pairs of correlated substitutions changing two binding energies such that the fitness remains constant. These lead to nucleotide frequency correlations between different sites. Such compensatory changes have indeed been observed in experiments on *Drosophila *promoters [44].

### Site-shadow interactions

In larger regulatory regions, there is a number of shadow sites where a binding sequence **a **would have a similar regulatory effect as at the functional sites present. In that case, the genomic functional element contains not only the functional binding sites but also the shadow sites. Once a functional site has disappeared due to deleterious mutations, a shadow site can turn functional by adaptive evolution as described in the last section. The resulting evolutionary dynamics leads to sequence turnover with the actual binding sites present at different but functionally equivalent positions [36]. Substantial sequence turnover has been observed in a number of case studies [44-49]. Also the number of actual sites is subject to evolutionary variation since the same regulatory effect, i.e., the same fitness, can be distributed over fewer stronger or more weaker sites. With increasing number L˜
 MathType@MTEF@5@5@+=feaafiart1ev1aaatCvAUfKttLearuWrP9MDH5MBPbIqV92AaeXatLxBI9gBaebbnrfifHhDYfgasaacH8akY=wiFfYdH8Gipec8Eeeu0xXdbba9frFj0=OqFfea0dXdd9vqai=hGuQ8kuc9pgc9s8qqaq=dirpe0xb9q8qiLsFr0=vr0=vr0dc8meaabaqaciaacaGaaeqabaqabeGadaaakeaacuWGmbatgaacaaaa@2DDC@ of shadow positions, one expects that the number of actual sites grows while individual sites get weaker [36].

### Gene interactions

Evolutionary interactions are not limited to regulatory elements for the same gene. An example are gene duplications and the subsequent evolution of the daughter genes. Selection acts jointly on this pair of genes [50], which have initially identical functions, eventually leading to either loss of one of them or to *subfunctionalization*, which has been argued to be an important mode of genome evolution in eukaryotes [51,52]. This process can take place by regulation, i.e., via a correlated distribution of the regulatory elements on the daughter genes. More generally, the evolution of genes in a regulatory network is correlated if their functions are coupled either in series (i.e., one gene acts on the other) or in parallel (i.e., they are part of alternative pathways for the same function). Although some regulatory networks in model organisms – e.g. the embryonic development in the sea urchin [53] – have been studied in detail, we lack a coherent view of their functional evolution to date.

### Interactions and time-dependent selection

The functional integration of regulation at multiple levels and the resulting fitness interactions (epistasis) imply that the selection at one genomic site is influenced by changes at other sites. A recent analysis of single-nucleotide polymorphisms and substitutions in *Drosophila *provides indeed evidence on a genome-wide scale that selection is time-dependent: at individual loci, changes in the direction of selection occur at nearly the rate of neutral evolution [54,55]. At the same time, selection is sufficiently strong so that the adaptive response can keep up with the rate of selection changes. This rate is faster in non-coding DNA, which points towards the role of regulation in the adaptive differentiation between species. Genomic evolution emerges as a complex stochastic process, shaped jointly be the driving force of time-dependent selection, fitness interactions between sites, and the ongoing background of near-neutral changes. Much more remains to be learned about the interplay of these evolutionary forces: in a large and strongly coupled system, one external signal can trigger an avalanche of subsequent compensatory responses. This dynamics seems now within reach of genomic sequence analysis.

### Evolutionary innovations

Under stationary selection, functional elements are more conserved than background sequence, and the score-fitness relation quantifies the amount of conservation. But evolution is, of course, not limited to conservation. On one hand, there is typically a multitude of different genotypes yielding the same molecular function, and the evolutionary dynamics continuously plays with these alternatives. On the other hand, organisms face long-term changes of their environment, which lead to new selection pressures and a response by adaptive evolution of *new functions*. If regulation is to account for a large part of the diversification in higher eukaryotes, loss or gain of regulatory function should be an important mode of molecular evolution. Changes in regulatory DNA leading to new functions of gene networks have been observed [56], and it is possible to extend the statistical models described in the previous section to include evolutionary gain or loss of function of individual binding sites [16]. On a broader scale, time-dependent selection and fitness couplings appear act as a major driving forces of genomic change, triggering avalanches of evolutionary innovation. Understanding this molecular basis of innovations is a major challenge for theory and experiment in the coming years. It will profoundly change our dynamical view of the genome.
